# Ecological success of extreme halophiles subjected to recurrent osmotic disturbances is primarily driven by congeneric species replacement

**DOI:** 10.1093/ismejo/wrae215

**Published:** 2024-10-23

**Authors:** Esteban Bustos-Caparros, Tomeu Viver, Juan F Gago, Luis M Rodriguez-R, Janet K Hatt, Stephanus N Venter, Bernhard M Fuchs, Rudolf Amann, Rafael Bosch, Konstantinos T Konstantinidis, Ramon Rossello-Mora

**Affiliations:** Marine Microbiology Group (MMG), Department of Animal and Microbial Biodiversity, Mediterranean Institute for Advanced Studies (IMEDEA, CSIC-UIB), 07190 Esporles, Spain; Marine Microbiology Group (MMG), Department of Animal and Microbial Biodiversity, Mediterranean Institute for Advanced Studies (IMEDEA, CSIC-UIB), 07190 Esporles, Spain; Department of Molecular Ecology, Max Planck Institute for Marine Microbiology, 28359 Bremen, Germany; Marine Microbiology Group (MMG), Department of Animal and Microbial Biodiversity, Mediterranean Institute for Advanced Studies (IMEDEA, CSIC-UIB), 07190 Esporles, Spain; Department of Microbiology, University of Innsbruck, 6020 Innsbruck, Austria; Digital Science Center (DiSC), University of Innsbruck, 6020 Innsbruck, Austria; School of Civil and Environmental Engineering, Georgia Institute of Technology, Atlanta, 30332 GA, United States; Department of Biochemistry, Genetics and Microbiology, and Forestry and Agricultural Biotechnology Institute (FABI), University of Pretoria, 0002 Pretoria, South Africa; Department of Molecular Ecology, Max Planck Institute for Marine Microbiology, 28359 Bremen, Germany; Department of Molecular Ecology, Max Planck Institute for Marine Microbiology, 28359 Bremen, Germany; Marine Microbiology Group (MMG), Department of Animal and Microbial Biodiversity, Mediterranean Institute for Advanced Studies (IMEDEA, CSIC-UIB), 07190 Esporles, Spain; Microbiologia, Departament de Biologia, Edifici Guillem Colom, Universitat de les Illes Balears, Campus UIB, 07122 Palma de Mallorca, Spain; School of Civil and Environmental Engineering, Georgia Institute of Technology, Atlanta, 30332 GA, United States; Marine Microbiology Group (MMG), Department of Animal and Microbial Biodiversity, Mediterranean Institute for Advanced Studies (IMEDEA, CSIC-UIB), 07190 Esporles, Spain

**Keywords:** osmotic disturbances, congeneric species replacement, viral cohort, metagenomics, time-series

## Abstract

To understand how extreme halophiles respond to recurrent disturbances, we challenged the communities thriving in salt-saturated (~36% salts) ~230 L brine mesocosms to repeated dilutions down to 13% (D13 mesocosm) or 20% (D20 mesocosm) salts each time mesocosms reached salt saturation due to evaporation (for 10 and 17 cycles, respectively) over 813 days. Depending on the magnitude of dilution, the most prevalent species, *Haloquadratum walsbyi* and *Salinibacter ruber*, either increased in dominance by replacing less competitive populations (for D20, moderate stress conditions), or severely decreased in abundance and were eventually replaced by other congeneric species better adapted to the higher osmotic stress (for D13, strong stress conditions). Congeneric species replacement was commonly observed within additional abundant genera in response to changes in environmental or biological conditions (e.g. phage predation) within the same system and under a controlled perturbation of a relevant environmental parameter. Therefore, a genus is an ecologically important level of diversity organization, not just a taxonomic rank, that persists in the environment based on congeneric species replacement due to relatively high functional overlap (gene sharing), with important consequences for the success of the lineage, and similar to the success of a species via strain-replacement. Further, our results showed that successful species were typically accompanied by the emergence of their own viral cohorts, whose intra-cohort diversity appeared to strongly covary with, and likely drive, the intra-host diversity. Collectively, our results show that brine communities are ecologically resilient and continuously adapting to changing environments by transitioning to alternative stable states.

## Introduction

How multiple rounds of the same or very similar disturbances affect microbial community stability and ecosystem function remains challenging to quantify and fully understand [1, 2]. Such recurrent disturbances (i.e. seasonal disturbances of temperature, humidity, light intensity) are frequent in natural ecosystems [3, 4] and have been shown to influence microbial assemblages by deterministic processes that trigger significant effects on species persistence and interactions. These fluctuations ultimately select for resistant organisms that confer “ecological resilience” to the ecosystem [1, 2, 5, 6]. By ecological resilience we refer to the ability of ecosystems to resist regime shifts and maintain functions, potentially through internal reorganization of species and their interactions, and the existence of multiple stable states [2]. The reorganization of species could involve, for example, the replacement of functionally redundant species with different responses to environmental perturbations [2]. Ecological resilience needs to be distinguished from “engineering resilience”, which refers to the speed with which the ecosystems return to an equilibrium state following disturbance [1, 2].

Within robust ecosystems, there are several successful species such as species of the genera *Prochlorococcus, Haloquadratum,* and *Salinibacter* that are capable of resisting environmental fluctuations (especially seasonal and/or recurrent disturbances) without apparent changes to their proportion and abundance. One explanation for this pattern is that such resistant species are comprised of distinct coexisting strains, each with distinct gene content and displaying niche partitioning, thus granting ecological success to face environmental fluctuations via strain replacement [7–11]. It remains less clear, however, if the same principle can be observed at a higher level; that is, replacement of (different) species of the same genus underlies the ecological success of many known genera in a wide range of environments.

As the study of specific ecological interactions in natural communities can be extremely complicated depending on the complexity of the ecosystem and the duration of the disturbance [12], mesocosms could be used to offer new insights into the abovementioned questions as long as they mimic well the natural condition. Among the model ecosystems to study community dynamics and diversity, solar salterns may be ideal to investigate how environmental disturbances affect inter- and intraspecies interactions and diversity [6, 9, 10, 13]. This is because, as we have previously demonstrated, saltern microbial communities show remarkable similarity between different sites in the world, independent as to if these are naturally occurring lakes or human-driven salterns [14, 15]. For the present study, the saltern communities show notable similarity even when the size of the ecosystem varies by several orders of magnitude, from natural lakes to small ponds and laboratory mesocosms, as shown previously [6, 10].

Solar salterns are semiartificial systems into which seawater is pumped to fill a series of interconnected ponds, and in which the salinity within these ponds increases over time due to evaporation. The different salts precipitate in distinct saturation stages, and in the most saturated ponds (crystallizers), NaCl precipitates at ~36% salinity, leaving magnesium salts still soluble [16]. Crystallizers are normally fed with preconcentrated brines ranging between 17% and 25% salts, and therefore the change in salinity when feeding the ponds is normally not very dramatic, but rather gradual. Only during heavy rain events, brines can experience stronger dilution. Extreme halophiles thrive in such crystallizers, but it is generally challenging for them to survive below 15% salts [17]. These hypersaline environments are widely distributed across the globe, and salinity and light intensity seem to be the main deterministic factors influencing the structure of their microbial and viral communities [6, 9, 10, 13]. It also seems that human intervention to construct crystallizer tanks for salt industrial production has selected for highly similar, but likely more robust to the recurring cycles of dilution and salt precipitation, microbial assemblages compared to populations in naturally occurring brines [18].

Brines close to salt saturation are composed of high densities of prokaryotic cells (>10^8^ cells/ml), with a relatively reduced species diversity, which are often accompanied by two orders of magnitude higher virus-like particles (>10^10^ VLPs/ml). These ecosystems are generally dominated by three major lineages, *Halobacteriota, Nanohaloarcheota,* and *Salinibacteraceae* [19–22], the representative genera of which, i.e. *Haloquadratum*, *Halorubrum,* and *Salinibacter*, may collectively reach >90% of the total community [22]. The difference in the relative abundance of these genera can be attributable to location-specific environmental conditions, and/or chemistry; even within the same location, adjacent ponds with similar salinities can show detectable (but not pronounced) differences in community structure [14]. The functional differentiation and ecological success of the species within these genera remain largely unknown, but it is reasonable to expect that high intraspecific genomic diversity and accompanying viruses allow differential adaption to the prevailing environmental conditions [9, 10, 21]. *Salinibacter* is a prominent example of a successful bacterial genus that always dominates the bacterial fraction of these systems, with *Sal. ruber* often, but not always, being the most abundant species of the genus [23]. In some cases, despite *Sal. ruber* being present, its abundance is surpassed by other members of the genus such as *Sal. abyssi* or *Sal. pampae* [23].

Here, we designed an 813-day-long experiment to reveal how recurrent osmotic cycles influence microbial and viral assemblages at the species and intraspecific level in communities of hypersaline brines. Specifically, we prepared two mesocosms consisting of a concentrated large volume (~230 L) of mature solar saltern salt-saturated brines (>36% salts) from crystallizer ponds that were repeatedly diluted, separately, to 13% (D13 mesocosm) and 20% (D20 mesocosm) salts each time salt concentration reached saturation conditions due to evaporation by natural sunlight and temperature. We performed shotgun metagenome sequencing of 130 samples from the two mesocosms and examined species and intraspecies diversity patterns.

## Materials and methods

### Mesocosms

**Figure 1:**
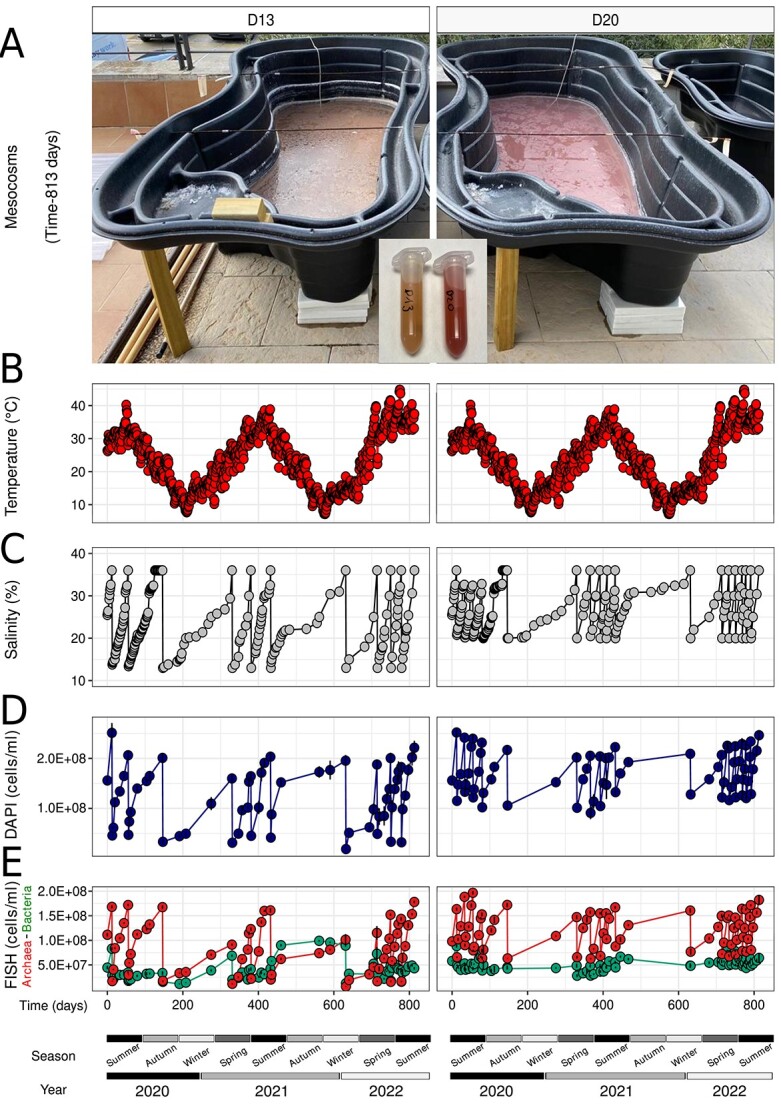
Experimental setup of the study. (A) Mesocosm appearance after 813 days, oscillations of (B) temperature (°C), (C) salinity (%), (D) total (DAPI), and (E) bacterial/archaeal (FISH) cell count dynamics (cells/ml) over the 813 days of long-term osmotic disturbance.

Two 1000 L plastic tanks (Fig. 1, Supplementary Figs S1 and S2) containing 230 L of salt saturated brines (~36% NaCl; [24]) obtained from the Salterns of S’Avall (39°19′28″N; 2°59′21″E) on 10 June 2020 were recurrently diluted to either 13% (D13) or 20% (D20) salts each time they reached salt saturation due to (natural) evaporation. To dilute the brines we used tap water (TW). The chemical and microbiological composition of TW is provided in Supplementary Text T1. The mesocosm operation lasted for 813 days with regular sampling (approximately monthly, but with higher frequency over the summer due to faster cycles of evaporation in that season) as indicated in Supplementary Text T1. To avoid undesirable dilution by natural rain, the tanks were shielded with transparent polycarbonate corrugated sheets placed above the tanks when the weather forecast indicated rain (Supplementary Fig. S2B). Please note that no biological replicates were available due to the large volume and frequent sampling of the mesocosms that render replication too costly, but also because replication is not necessary in time-series experiments like ours where microbial community variability is relatively small according to recent literature [25, 26].

### Ionic composition, total cell counts, and fluorescence *in situ* hybridization

Brines were diluted 1:400 and filtered through 0.22 μm hydrophilic PTFE filters, and the filtrate was sent to the Technical Research Services of Alicante University, Spain for ionic characterization. The ions were quantified by ion chromatography as previously described [15, 23]. Samples were immediately fixed with formaldehyde and processed using standard protocols to calculate cell numbers using fluorescence microscopy [27]. Total cell counts were determined using DAPI and the archaeal (ARCH915) and bacterial (EUB338) fractions determined using domain-specific probes for fluorescence *in situ* hybridization or FISH [27].

### DNA extraction, sequencing, and metagenomic analyses

As previously detailed [28], 25 ml of brines were used for DNA extraction. Samples were sequenced on a HiSeq System (Illumina; 2 × 150 bp, paired-end reads) at Macrogen, South Korea. Raw reads were trimmed using bbduk v38.82 [29] (quality score ≥ 20 and length ≥ 100 bp). Trimmed reads were assembled using MEGAHIT v1.2.9 [30] and metaSPAdes v3.14.1 [31], and only contigs with length ≥ 500 bp were kept. Metagenomic statistics are summarized in Supplementary Table S1. Prodigal v2.6.3 [32] was used to predict genes encoded in assemblies. Predicted proteins were annotated using GhostKoala v2.0 tool [33] with default parameters. The abundance of each non-redundant gene across samples was determined by read mapping using Bowtie2 v2.3.4.1 [34], followed by best-match counting only when higher than 95% identity as implemented in samtool v.1.10 and bedtools v2.30.0 [35, 36]. Reads were assigned to KEGG Orthology (KO) based on their best-matching gene, similar to our previous studies [37]. 16S rRNA gene sequences were extracted and classified in Operational Phylogenetic Units (OPUs) as indicated in Supplementary Text T2.

### Binning, de-replication of MAGs in species, phylogenetic inference, and taxonomic classification

Binning was performed with MetaBAT2 [38] and MaxBin v2.1.1 [39] using contigs with length ≥ 2000 bp. MAGs with estimated completeness ≥50% and contamination <10% based on CheckM2 v0.1.3 analysis [40] were kept for further analyses. Dereplication of MAGs at the species level was conducted within and between metagenomes using dRep v3.2.0 [41] based on a FastANI algorithm with a cut-off of Average Nucleotide Identity (ANI) value ≥95%. Next, dRep clusters based on FastANI values were manually checked using the script *ani.rb* from the enveomics collection [42], to corroborate that the MAGs represented the same species (ANI ≥ 95%) [43, 44]. Representative MAGs for each resulting species were assigned based on the highest quality score (Completeness − 4 × Contamination). The phylogenetic reconstruction based on single-copy core genes was performed as previously detailed [23]. The representative MAGs were taxonomically classified with GTDB-Tk v2.1.1 tool [45] using the release207_v2.

### Viral contig identification, taxonomic classification, and clustering in viral OTUs (vOTUs)

To ensure accurate identification, taxonomic classification, and prediction of lytic and lysogenic viruses, contigs ≥5000 bp were extracted from metagenome assemblies and subjected to geNomad [46] analysis. The identified viral contigs were clustered in vOTUs at ≥95% ANI with at least ≥85% coverage relative to the shortest contig using CD-HIT v4.8.1 [47] (parameters: -c 0.95 -aS 0.85), selecting the longest sequence as representative of a given vOTU in subsequent analyses.

### Relative abundances of species and vOTUs

Relative abundances were estimated using a representative MAG of each species and the representative sequence of each vOTU across metagenomes. For this, the trimmed reads were mapped to contigs using Bowtie2 v2.3.4.1 [34] and best-match mapped reads were filtered at 95% identity with samtool v.1.10 and bedtools v.2.30.0 [35, 36]. The sequencing depth of mapped reads was further normalized by employing a truncation to the middle 80% strategy (i.e. removing the upper and lower 10% positions by depth) by the use of the *BedGraph.tad.rb* script of the enveomics collection [42]. ANIr, the average nucleotide identity of mapped reads against a reference genome, was calculated using the *anir.rb* script [42]. The average genome size was estimated using the MicrobeCensus tool [48]. Average genome size was similar across metagenomes; hence, further normalization of abundance values was not necessary. Then, to compare microbial and viral species abundances across metagenomes, sequencing depth was divided by the total number of reads in the metagenome and multiplied by 10^8^ (the sequencing effort). Functional annotation of the MAGs was carried out using the COGclassifier tool v.1.0.5 (https://github.com/moshi4/COGclassifier/) with default parameters.

### Metabolic redundancy of species within different genera

Metabolic redundancy within a genus was analyzed using both all type strain genomes publicly available as well as (separately) our collection of MAGs of *Haloquadratum*, *Halorubrum,* and *Salinibacter* genera with ≥70% completeness and <5% contamination. For this, proteins of each genome/MAG were predicted using Prodigal v2.6.3 [32] and proteins shorter than 100 aa were removed from the analyses. The resulting proteins were clustered using CD-HIT v4.8.1 [47] (parameters: -c 0.5 -aS 0.5 -n 3) and functionally annotated using MicrobeAnnotator v.2.0.5 [49] with default parameters. Only KEGG modules with ≥50% of completeness were kept for metabolic redundancy analyses.

### Viral-host assignation and clustering in viral cohorts

Specific viral-host linkages were predicted using iPHoP v1.3.0 [50] with default parameters and score ≥75 [51]. The viral-host assignments were carried out selecting the putative host (including MAGs recovered from our dataset and from the available genomes from NCBI) with the highest iPHoP score obtained. vOTUs assigned to the same host were grouped in “*viral cohorts*” as long as their relative abundance across samples correlated strongly based on the extended Local Similarity Analysis (eLSA) technique, with the following cut-off: *LS* > 0.6 and *P* < .05 [52]. Significant correlations were employed to group the vOTUs using a Markov Clustering Algorithm (MCL), as previously reported [53].

### Statistical analyses

Alpha-diversity based on OPUs, vOTUs, and KOs was predicted using *AlphaDiversity.pl* script from the enveomics collection [40]. Beta-diversity statistical analysis based on (i) MASH distances [54], (ii) MAGs, and vOTU relative abundances was conducted in R v4.1.2 [55]. Bray–Curtis dissimilarity matrix, Non-metric multidimensional scaling (NMDS), and Procrustes based on microbial and viral relative abundances were performed using the vegan package in R [56]. Differential abundance of microbial and viral genomes was assessed by LefSe tool v1.1.2 [57]. Linear Discriminant Analyses (LDA) > 2 and *P* < .05 were considered significant.

## Results

### Mesocosm composition and growth condition overview

In total, 640 L of brines at 25.5% salts were split in two 320 L mesocosms and concentrated to ~230 L each, which resulted in salt saturation conditions (>36% salts) as shown in Supplementary Text T3. Before the first dilution event (22 June 2020), both mesocosms were salt-saturated and contained ~2.52 × 10^8^ ± 8.5 × 10^6^ cells/ml with the fraction of archaeal vs bacterial cells at ~66% and ~34%, respectively (Fig. 1D and E; Supplementary Table S1). Metagenome, microscopy, and ionic composition analyses of the tap water (TW) used to dilute brines indicated a negligible influence of the TW prokaryotic or eukaryotic cells and ions, including any DNA released from cell lysis (TW contained <8.5 × 10^6^ cells/ml, i.e. at least two orders of magnitude fewer cells in TW; Supplementary Text S4 and Supplementary Fig. S3; Supplementary Fig. S4). Temperatures did not differ between mesocosms ranging from a low level of ~5.3°C (winter) to a high level of ~45.6°C (summer) (Fig. 1B; Supplementary Table S1). The D13 mesocosm, in which the salinity was reduced to 13% salts, was subjected to a total of 10 dilution-evaporation cycles (Fig. 1C; Supplementary Table S1), and ~316 L of TW was added at the end of each cycle for the dilution step. During spring–summer periods, the duration of the cycles was shorter, with an average duration of 52 ± 23 days, and 2, 2, and 3 full cycles having taken place in years 2020, 2021, and 2022, respectively (Fig. 1B and C; Supplementary Table S1). During autumn–winter, the duration of the cycles was substantially longer, averaging 192 ± 11 days, with 1 and 2 cycles occurring in 2020 and 2021, respectively (Fig. 1C; Supplementary Table S1). The salinity in the D20 mesocosm was reduced to 20% salts by the addition of ~139 L of tap water at the end of each cycle. During the spring–summer periods, there were 4, 4, and 6 cycles in years 2020, 2021, and 2022, respectively (Fig. 1C; Supplementary Table S1) with an average of 24 ± 14 days per cycle, whereas during the autumn–winter period the number of cycles was identical to those of D13.

### Evaporation-dilution events select for resistant archaeal and bacterial species

After 813 days, the effect of distinct dilution regimens resulted in diverging color of the two brines (Fig. 1A; Supplementary Fig. S1D), indicating distinct microbial community dynamics. The evaporation-dilution cycles resulted in a decrease in total cell counts, which was more pronounced in winter, with a reduction of 1.15 and 1.34-fold in cell concentrations for the D20 and D13 mesocosms, respectively (Fig. 1D; Supplementary Table S2). In D20, archaeal cells (73.57% ± 4.89% of total) were dominant throughout the duration of the experiment (Fig. 1E; Supplementary Table S2). In contrast, in D13, the absolute abundance of Archaea was reduced, which was accompanied by a substantial increase of Bacteria after each dilution event, and this was more pronounced during the colder seasons than in the warmer seasons. Bacteria surpassed Archaea in abundance during the second winter period in D13 reaching, after 435 days since the start of the operation of the mesocosms, 66% of the total microbial population.

Total cell count analysis showed that the first dilution event alone resulted in a 2.2-fold reduction in total cells/ml in D20 (from 2.51 × 10^8^ ± 1.95 × 10^7^ to 1.2 × 10^8^ ± 3.4 × 10^6^ cells/ml), diverging from the expected 1.8-fold reduction solely by the dilution itself (Fig. 1D; Supplementary Table S2). FISH quantification showed that these discrepancies were mainly attributable to a 2.5-fold reduction of archaeal cells (from 1.63 × 10^8^ ± 2.9 × 10^6^ to 6.57 × 10^7^ ± 1.5 × 10^6^ cells/ml), contrasting with a 1.8-fold decrease observed for bacterial cells (from 8.83 × 10^7^ ± 2.9 × 10^6^ to 4.49 × 10^7^ ± 1.5 × 10^6^ cells/ml) (Fig. 1E; Supplementary Table S2). A much stronger effect occurred in D13, in which the first dilution induced a 5.5-fold reduction of cells (from 2.51 × 10^8^ ± 1.95 × 10^7^ to 4.6 × 10^7^ ± 2.3 × 10^6^ cells/ml), or nearly twice as pronounced as the expected 2.8-fold decrease after dilution (Fig. 1D; Supplementary Table S2). The cell reduction in D13 was 9.9-fold for Archaea (from 1.68 × 10^8^ ± 2.8 × 10^6^ to 1.7 × 10^7^ ± 5.9 × 10^5^ cells/ml) and only 2.9-fold for Bacteria (from 8.3 × 10^7^ ± 2.8 × 10^6^ to 2.89 × 10^7^ ± 5.9 × 10^5^ cells/ml) (Fig. 1E; Supplementary Table S2). Therefore, a more pronounced cellular lysis was observed for Archaea than for Bacteria. However, ratios of lysed vs. intact cells declined over time indicating a selection of populations (or mutations) that were more resistant to the recurring osmotic shocks (Supplementary Fig. S5; Supplementary Table S2). Between the initial and final mesocosm samples, osmotic shock-resistant bacterial cells, as assessed by the visualization of intact cells under the microscope by a domain-specific FISH probe, increased from 34.9% to 40.2% in D13 and 55.9% to 77.6% in D20. A similar pattern was observed for the shock-resistant Archaea increasing from 10.1% to 11.5% in D13 and from 40.2% to 46.8% in D20. Curiously, after the longer winter cycles, we detected a notable increase in mortality after the first dilution compared to dilutions as part of the summer cycles, attributable apparently to the increase of shock-sensitive cells during the longer cycles of the colder periods.

### Recurrent dilution constantly drives the selection of adapted members of the communities

**Figure 2:**
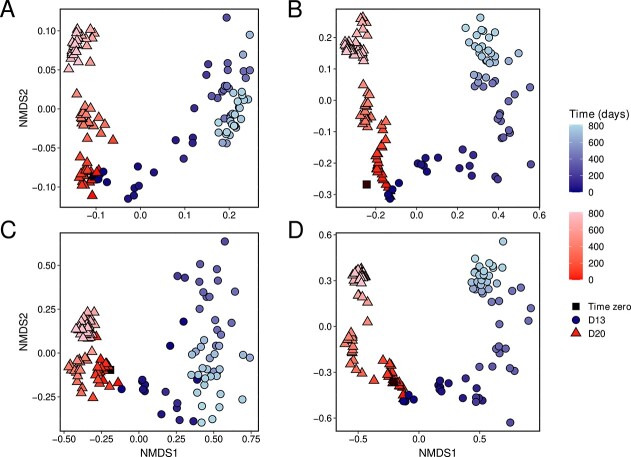
Temporal dynamics of prokaryotic and viral communities over 813 days of disturbance. Non-metric multidimensional scaling (NMDS) performed on (A) MASH distances. NMDS using Bray-Curtis dissimilarities based on relative abundances of (B) MAGs, (C) OPUs, and (D) vOTUs. Symbol shapes represent the mesocosm of origin for the samples, while the gradient indicates temporal succession. Black squares indicate time zero (10 June 2020).

Nearly identical microbial and viral composition of both D13 and D20 at time zero (whole-metagenome MASH distance value of 0.026; Fig. 2; Supplementary Table S3) diverged during the experiment. After the first three dilution events, D20 samples displayed substantial similarity among themselves with MASH distances ranging from 0.026 to 0.033. The longer evaporation period in winter promoted a significant shift (MASH value increased from 0.042 to 0.061), and this trend repeated in the following years and was mirrored by the cell counts. During the spring–summer periods, the communities did not show large changes in their composition, which change more drastically during the autumn–winter periods. In contrast, D13 showed greater differentiation after the first two dilution events, and contrary to D20, the D13 community transitions were continuous, reaching a stable composition after ~700 days (Fig. 2; Supplementary Table S3).

### Coupled microbial and viral community dynamics throughout dilutions

From the 130 sequenced metagenomes, we recovered a total of 2745 MAGs and 38056 putative viral contigs, rendering 128 species and 7451 vOTUs, respectively, after de-replication. From the binned MAGs, some were recovered from samples with coverages as low as 0.02%, but still with relatively good completion (64%) and low contamination (3.45%) or 0.05% with 61% completion and 9.9% contamination, as was the case of *Halomicrobium* sp2 (Supplementary Tables S4 and S8).

The 99.48% of the vOTUs were predicted to be lytic viruses, and 47.82% of them were assignable to 97 microbial species, indicating putatively active viral predation (Supplementary Text T5; Supplementary Tables S4–S7). Procrustes analyses using relative abundances of individual MAGs (genomes, Fig. 2B) or OPUs (16S rRNA variants; Fig. 2C) against the detected vOTUs (viruses; Fig. 2D) showed significant correlation (values ranging from 0.75 to 0.97; *P* < 0.01). These reflected strong interdependencies of prokaryotic and viral communities in terms of their species diversity, which were further corroborated by strong similarity in MASH distance dynamics (Fig. 2A).

### Intense dilution shapes the microbial community structure

**Figure 3:**
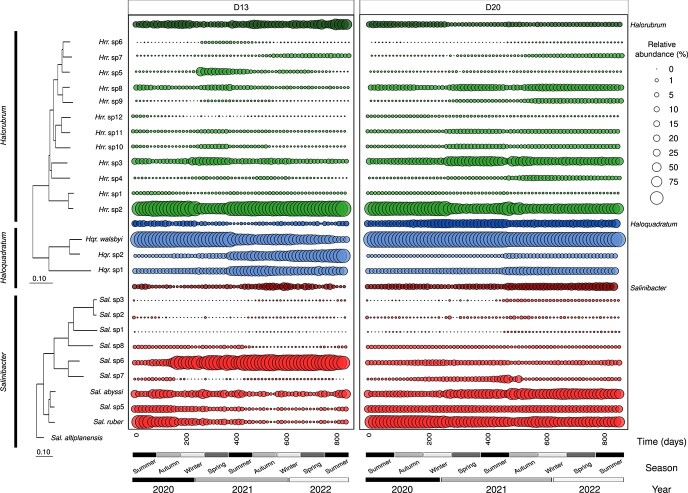
Shifts of species abundance and interspecific genus replacement. Relative abundance of *Halorubrum*, *Haloquadratum,* and *Salinibacter* species in the D13 and D20 metagenomes (*n* = 130) was estimated using sequencing effort (sequencing depth divided by number of metagenomic reads). Darker bubbles show the sequencing effort of each genus across the metagenomic dataset, while the lighter bubble represents the relative abundance of each species within each genus. Phylogenetic reconstruction using the concatenated core orthologous proteins of *Halorubrum, Haloquadratum,* and *Salinibacter* species extracted from our experiment. Both phylogenetic trees were reconstructed with a neighbor joining algorithm using the Kimura correction. The bar indicates the 10% sequence divergence in each tree.

Microbial diversity indices based on MAGs, OPUs, and vOTUs are described in Supplementary Text T5. The original microbial composition was consistently dominated by *Hqr. walsbyi*, *Sal. ruber,* and diverse *Halorubrum* species, with a special dominance of the yet uncultured *Halorubrum* sp2 (Fig. 3; Supplementary Table S8; Supplementary Fig. S6). In D20, the Linear Discriminant Analysis (LDA) revealed consistent dominance of *Haloquadratum* and *Salinibacter* species throughout the experimental period (LDA > 2; *P* < .01; Supplementary Fig. S7), with a notable gradual increase in the relative abundance (or just abundance from now on for metagenome-based data) of the uncultured *Sal. abyssi,* eventually reaching comparable levels with the dominant *Sal. ruber* at later stages (Time-813 days: 6.32%; Fig. 3; Supplementary Table S8). To a lesser extent, we also detected an increase in the abundance of another uncultured *Sal.* sp5 (Time-813 days: 3.26%). The increase in abundance in the latter two *Salinibacter* species coincided with a decrease of the initially abundant *Halorubrum* sp2. A notable increase in the abundance of *Halonotius* sp2 was also observed after the second winter dilution event with an abundance from 0.25% at time zero to 2.72% at time-813 days (Supplementary Fig. S6; Supplementary Table S8).

An opposite trend was observed for D13, for which the two dominant species, *Sal. ruber* and *Hqr. walsbyi*, declined substantially in abundance (Fig. 3; Supplementary Table S8; Supplementary Fig. S6). In D13, the initially abundant *Halorubrum* sp2 further increased in abundance, which contrasted with its significant decrease in D20. In D13, the dilution events seemed to create a favorable environment for *Halorubrum* sp2, becoming the predominant species at later stages of the mesocosm with an abundance of 30% of the total community, which exceeded the combined sum of all other species with binned MAGs available. Other species of *Halorubrum*, *Halobellus*, and various members of the uncultured archaeal genera (such as A07HB70 and CBA1134) exhibited a marked preference for broader salinity and temperature ranges (as indicated by LDA > 2; *P* < 0.01; Supplementary Figs S6 and S7). The decrease of *Sal. ruber* during the first winter paralleled the emergence of *Sal.* sp6, which surpassed the abundance of *Sal. ruber* (Time-715 days: 18.75% of the total community). A similar trend was observed for *Hqr. walsbyi*, for which the decline of this species was accompanied by the emergence of *Hqr.* sp2 (Fig. 3; Supplementary Fig. S6). The notable taxonomic shifts observed in D13 were accompanied by a significant reduction in average species diversity within the archaeal domain and a parallel strong increase of the bacterial counterpart (Supplementary Fig. S8). This increase was especially apparent after the first long winter dilution event and was attributable to the species affiliated with *Bacteroidota, Pseudomonadota, Cyanobacteria,* and *Verrucomicrobiota* (Supplementary Table S8), and was accompanied by an increase in functional diversity (Supplementary Fig. S9B).

In both mesocosms, the MAGs binned for many of the 128 species detected were of rare occurrence as these appeared in abundances <0.1%, and in many samples no recruitment was possible as their abundance was below the detection limit (Supplementary Table S8 and Supplementary Fig. S6). Often, these very low abundance species appeared sporadically, and never maintained a constant detectable abundance in the system.

### Functional redundancy ensures the ecological success of genera

Our time-series showed that when disturbances did not cause dramatic shifts in the level of stress, the coexisting species of the same genus exhibited similar dynamics (e.g. D20 mesocosm). However, when adverse conditions negatively affected one or several species, one of their congeneric, but not necessarily the closest-relatives, seemed to replace their ecological space, especially in the D13 mesocosm. The latter trend appeared to be common in various abundant genera of the community such as *Haloquadratum, Halorubrum,* and *Salinibacter* (with 3, 9, and 12 distinct species, respectively), and 8 out of 14 additional genera represented by 2 or more species (Fig. 3 and Supplementary Fig. S10). The genera for which we did not see clear replacement were generally found in low abundance, and thus their members might have been under the limit of detection of our sequencing effort. As the replacement is likely due to a higher ecological fitness (e.g. emerging species withstanding a greater change in salinity in D13; Fig. 3), we compared the pangenomes of distinct species of the genera *Haloquadratum, Halorubrum,* and *Salinibacter* to obtain insights into functional redundancy and uniqueness. For this, we used all publicly available type-strain genomes as well as our MAG collection to evaluate differences in the core (i.e. present in >90% of type-strain genomes) and auxiliary gene repertoire. For *Halorubrum* and *Salinibacter*, we found that type-strain genomes shared 51.73 ± 4.02% and 72.87 ± 1.52% of their proteins, respectively (Supplementary Table S9), which presumably reflected the larger phylogenetic divergence of *Halorubrum* species relative to species of *Salinibacter* (i.e. higher genetic/phylogenetic distance typically corresponds to higher gene-content differences; Supplementary Fig. S11A and B). The MAGs of the same genera showed consistent patterns overall with those based on type isolate genomes; that is, the *Halorubrum* and *Salinibacter* MAGs shared 41.78 ± 3.04% and 71.28 ± 5.27% of their proteins, respectively, with type-strain redundant proteins (Supplementary Table S9). *Haloquadratum* genomes revealed an even higher gene functional relatedness with 85.95% ± 11.78% of proteins shared between the three species of the genus (Supplementary Table S9). Functional annotation revealed that the core protein repertoire of each genus was principally related to the main carbon pathways (e.g. glycolysis or TCA cycle), carbohydrate, cofactor, vitamin, and amino acid metabolism, and ATP synthesis (Supplementary Fig. S11C), i.e. the central metabolic pathways expected for such heterotrophic organisms.

Main gene-content differences between species of the same genus were found in the auxiliary gene repertoire as opposed to the core genes. For instance, *Hqr.* sp2 encoded for higher content of both organic and inorganic transporters, cell control, transcription, translation, signal transduction, cell membrane biogenesis (e.g. glycosyltransferases) proteins, defense, and mobile elements compared to *Hqr. walsbyi* that it seemed to replace in D13 (Supplementary Fig. S12; Supplementary Table S10)*.* Similarly, *Sal.* sp6, which surpassed *Sal. ruber* in abundance at later sampling points in D13, contained a larger set of genes related to cell membrane biogenesis, mainly glycosyltransferases, replication, recombination and repair, signal transduction mechanisms, and intracellular secretion functions than *Sal. ruber* (Supplementary Fig. S12; Supplementary Table S10). These auxiliary genes may have played a role in the different dynamics observed, although adaptive mutations in one or a few of the many shared core genes (≥52% of the total genes in the genome) could not be excluded as an alternative explanation. These dynamics reveal that closely related species may occupy similar ecological spaces, but their distinct fitness toward changing environments may lead to species switching with no apparent significant changes in abundance at the genus level.

### Viral cohort dynamics differ between the two mesocosms

To evaluate the dynamics of the viruses in the system, and especially changes in the viral-host abundance ratios (VHRs), we focused on 57 microbial species each with 10 or more assigned vOTUs (Supplementary Table S7). On average, viral abundances clearly exceeded microbial abundances (VHRs >1; Supplementary Figs S13 and S14), indicating that a substantial portion of the viral communities was putatively infecting their host. Consistently, ~99.5% of the total vOTUs recovered were predicted to have a lytic lifestyle. The conditions generated in the D13 mesocosms clearly resulted in increased VHRs compared to the conditions in the D20 mesocosm (*P* < 0.01; Supplementary Fig. S14A), especially for viruses putatively infecting archaeal species (*P* < 0.01; Supplementary Fig. S14C).

Further, we clustered the vOTUs putatively infecting the same host and showing a common abundance pattern (Supplementary Fig. S15) into “viral cohorts” (Supplementary Table S11) based on the assumption that identical patterns should indicate the same infecting populations [58]. Specifically, we selected the viral cohorts associated with the key species *Hqr. walsbyi, Hqr.* sp2, *Sal. ruber,* and *Sal.* sp6 for further analyses. We identified from 70 to 522 vOTUs associated with each species (Supplementary Table S7) that were clustered into 4, 14, 19, and 23 distinct viral cohorts for *Sal. ruber*, *Sal.* sp6, *Hqr.* sp2*,* and *Hqr. walsbyi,* respectively (Supplementary Table S11).

**Figure 4:**
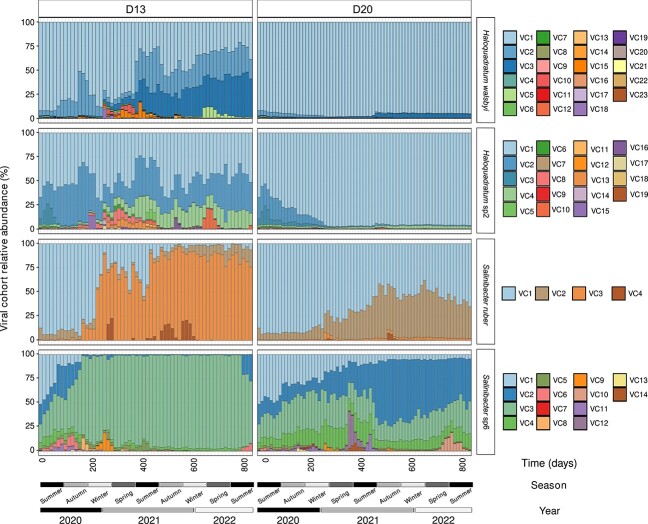
Dynamics of viral cohorts (VCs) predicted to infect *Hqr.walsbyi*, *Hqr.* sp2, *Sal.ruber*, and *Sal.* sp6. Relative abundances were calculated based on sequencing effort normalized by the total abundance of viral cohorts putatively infecting the same host across the metagenomes (*n* = 130).

We did not observe relevant changes in the relative abundance of the dominant viral cohorts or cohort replacement for *Hqr. walsbyi* and *Hqr.* sp2 in D20. For these cohorts, we observed instead a stable and increasing dominance of their respective and initially dominant Hqrw_VC1 and Hqrsp2_VC1 viral cohorts (Fig. 4). In contrast, in D13 the same two initially dominant viral cohorts declined over the course of the experiment, but still remained dominant. This reduction in abundance was accompanied by an increase of other viral cohorts, indicating an increase of the intraspecific diversity of each host. For *Sal. ruber* and *Sal.* sp6, the D20 conditions favored the increase of viral intra-cohort diversity over time, primarily by reducing the abundance of the dominant Salr*_*VC1 and Salsp6_VC1 viral cohorts, and enhancing the abundance of other coexisting viral cohorts (Salr*_*VC2 and Salsp6_VC2; Fig. 4). In D13, changes in the viral intra-cohorts were more pronounced and the low abundance cohorts Salr_VC3 and Salsp6_VC3 greatly increased and almost replaced the rest (Fig. 4). Collectively, these data suggested that the increase of viral cohort diversity was accompanied by (or driving) the shift of host intraspecific diversity. We also noted a peak of ephemeral viral cohorts, that likely infected archaeal or bacterial populations, which increased during the winter cycles, but probably declined over the short summer cycles.

### Viral cohort diversity strongly correlates with host intraspecific diversity shifts

**Figure 5:**
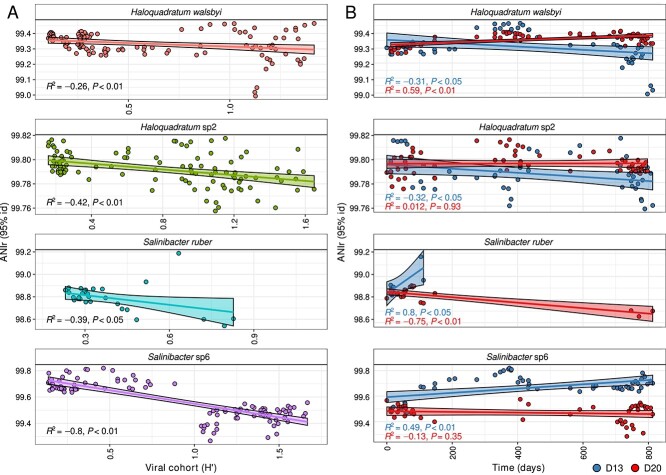
Correlation between viral cohort diversity and intraspecific microbial population shifts (ANIr). (A) Pearson’s correlation between the ANIr of MAGs mapped back to their source metagenome versus the Shannon index of the viral cohorts infecting *Hqr. walsbyi, Hqr.* sp2, *Sal. ruber,* and *Sal.* sp6. (B) Pearson’s correlations of ANIr of MAGs mapped back to their source metagenome for *Hqr. walsbyi, Hqr.* sp2, *Sal. ruber,* and *Sal.* sp6 over time in D13 and D20 mesocosms.

Intraspecific dynamics of the host are reflected by changes in the ANIr (mean of the sequence identity of all reads mapping on a MAG with at least 95% nucleotide identity that represent the same population as the MAG), where an increase in ANIr reflects a decrease in sequence diversity and a more clonal population. The key host species *Hqr. walsbyi, Hqr.* sp2*, Sal. ruber,* and *Sal.* sp6 (Supplementary Table S4) showed shifts in ANIr that significantly correlated with shifts in diversity of their respective viral cohorts (Pearson’s *R*^2^: −0.8 to −0.26, *P* < 0.01; Fig. 5A). Specifically, for D13, the populations of both host species *Hqr. walsbyi* and *Hqr.* sp2 showed significantly decreased ANIr values over time from 99.3% to 99.02% and 99.8% to 99.76%, respectively (*P* < 0.01; Fig. 5B), and a corresponding reduction in the abundance of the respective infecting viral cohorts Hqrw_VC1 and Hqrsp2_VC1 (Fig. 4). In parallel, other viral cohorts that were originally less abundant increased in abundance. D20 showed the opposite trend for both species, i.e. ANIr values further increased (*P* < 0.01; Fig. 5B) with an increase of the already dominant Hqrw*_*VC1 and Hqrsp2_VC1 cohorts, thereby displacing other less abundant viral cohorts such as Hqrw*_*VC2, Hqrsp2_VC2, and Hqrsp2_VC3 and resulting in a loss of the cohort’s diversity. Despite the fact that we did not obtain MAGs in all D13 samples for both *Salinibacter* species due to their relatively low abundance in these samples, we could observe several consistent trends. *Sal.* sp6 significantly increased in ANIr values from 98.74% to 98.93% and 99.55% to 99.7%, respectively (*P* < 0.01; Fig. 5B), which was presumably driven, to a certain extent, by an increase in dominance of Salr_VC3 and Salsp6_VC3 (Fig. 4). Contrarily, in D20 the ANIr of hosts *Sal. ruber* and *Sal.* sp6 decreased significantly from 98.74% to 98.6% and 99.55% to 99.44%, respectively (*P* < 0.01; Fig. 5B), reflecting an increase in abundance of the Salr_VC2 and Salsp6_VC2 viral cohorts (Fig. 4).

## Discussion

Lowering salinity to a minimum of 20% salts apparently resulted in a highly stable system that was not osmotically challenging for the well-adapted (to salt saturation conditions) extreme halophilic species in the original salt-saturated brines. For instance, the D20 mesocosm showed an increase in abundance of the well-known successful (in solar salterns) species, *Hqr. walsbyi* and *Sal. ruber*. In contrast, the dilution to 13% salts obviously exceeded the lower survival limit of 15% for the extreme halophiles [17]. For example, *Sal. ruber* cultures are incapable of growth below 15% salts [59], and *Hqr. walsbyi* cultures, despite maintaining viability and some growth at 12% to 14% salts, show optimum growth above 18% salinity [60]. Many extreme halophiles and especially Archaea are sensitive to extreme salinity changes that cause substantial cell mortality either due to mechanic cell disruption through osmotic shock or the activation of the infecting viruses [10, 61, 62]. The dilution to 13% salts promoted the increased abundance of distinct and less abundant species of Archaea and moderate halophilic Bacteria, which apparently prefer lower salinities and still remain viable at higher salinities [10].

Our time-series metagenome sequencing showed that a more extreme disturbance (D13 mesocosm) caused the communities to lose their equilibrium and transition to a new equilibrium, which was more different from time zero in the D13 mesocosm compared to the D20 mesocosm. In this new equilibrium in D13, the most abundant species initially in the brine was displaced by other species that were apparently more resistant to the environmental perturbations [1, 2]. The newly abundant species tended to be members of the same genus as the original prevalent species, e.g. the dominant species *Hqr*. *walsbyi* and *Sal. ruber* were largely replaced by the closely related species *Hqr.* sp2 and *Sal.* sp6. No members of other genera became abundant during our almost 3-year-long incubation. The maintenance of the genus level population structure by coexisting species of the same genus may explain the general dominance of *Haloquadratum, Salinibacter,* and *Halorubrum* genera in salterns around the globe and their resistance to extinction in a system undergoing frequent osmotic pressure changes [10, 63]. In accordance with the hypothesis of replacement within an ecosystem facilitated by relatively high (but not complete) functional redundancy [1, 2], the metabolic redundancy within these species, especially in terms of central carbon metabolism and resistance mechanisms to osmotic stresses, was high (e.g. 70%–80% of total genes in the genome were often shared among these species). This functional redundancy likely explains the observation that when *Sal. ruber* did not dominate, another congeneric species such as *Sal. abyssi, Sal. grassmerensis,* or *Sal. pampae* did dominate [23].

Consistent with functional redundancy, the most diverse genus detected in the system, the *Halorubrum* [64], deviated the most from the main replacement patterns described. Specifically, there were 11 coexisting *Halorubrum* species detected*,* but one of them (*Hrr.* sp2) maintained dominance throughout the course of the experiment in both mesocosms, being the most robust and best adapted species from the beginning. The only deviation to this pattern was an ephemeral congeneric species replacement in D13 during the first winter period that reverted to proportions similar to the initial stages when the temperatures rose again. In this case, perhaps salinity was not the main factor responsible for the replacement, but the temperature shift or a combination of both. However, in D20, *Hrr.* sp2 maintained its dominance, but was also accompanied by another six *Halorubrum* species that clearly increased in abundance during the incubation. Altogether we detected 128 distinct species, some binned with abundances as low as 0.02%, but still with good completion (64%) and low contamination (3.45%), and that may be part of the rare biosphere (less than 0.1% relative abundance) [65]. Many of these were sporadically detected with abundances just above the detection limits. However, the depth of sequencing and the use of short-read sequencing excluded an in-depth analysis of the interesting topic of changes in the rare biosphere induced by the salinity perturbation schemes applied here. This will be the topic of future studies.

Collectively, these results provide support for the ecological, and thus biological, significance of the taxonomic category of genus. It seems that this category has a true ecological meaning, which is driven, at least in part, by the higher gene content similarity of related species at this level compared to higher taxonomic ranks (e.g. > 50% of genes in the genome are shared among species of the same genus). Over the many years of molecular ecological studies, the coexistence of congeneric species with different gene content and niche specialization has previously been described for marine genera such as *Prochlorococcus* [7] or terrestrial genera as *Curtobacterium* [66], not to mention the studies on the coexistence of different *Salinibacter* species in the same brine [14, 23]. However, this study is documenting dynamics of congeneric species replacement in response to changes in environmental or biological conditions (e.g. phage predation) within the same system and with a controlled relevant environmental perturbation. Also, this study provides both a finer-resolution taxonomic study of the responses of 17 coexisting genera to environmental transitions as well as it documents several examples of clear intrageneric species replacement that mediate—at least in part—these responses.

The novelty of the study is in using a highly controlled multiannual perturbation on a complex, “close to natural” microbial community, to show species replacement within multiple genera based on metagenomics. By assigning ecological relevance to the taxonomic category of genus, our findings have important implications for molecular ecology studies. Our results suggest that the numerous 16S rRNA gene amplicon surveys identifying partial sequences down to (typically) the genus level should be trusted with higher confidence in terms of providing both taxonomically relevant information and rough metabolic and ecological predictions, as desired when studying core microbiomes [67]. Furthermore, our results could explain the success of many key genera in a wide range of environments, encompassing marine (e.g. *Prochlorococcus*) [7], freshwater (e.g. *Polynucleobacter*) [68], soil (e.g. *Curtobacterium*) [66], fish gut (e.g. *Ralstonia*) [69, 70], geothermal (e.g. *Venenivibrio*) [71], mammal gut (e.g. *Blautia*) [72], legume root nodules (e.g. *Micromonospora*) [73], or the hypersaline ecosystems studied here. That is, the success of a genus in a given ecosystem may be related to the diversity of its member species, in the same way as species’ success may depend on the intraspecific genetic diversity of the coexisting genomovars or strains [9, 10, 13]. Perhaps the success of one species does not only depend on its pangenomic repertoire, but also on the success of the coexisting congeneric species in a changing environment, a hypothesis that should be experimentally tested in the future.

As previously speculated, coexisting and (partially) functionally redundant microorganisms may differ not only in their physiological performance, but also in other traits influencing growth under specific conditions [74], resulting in divergent responses to environmental perturbations [1, 2]. As the genetic repertoire of the congeneric species seems to be largely functionally redundant, at least for the major metabolic and energy generating pathways, we speculate that only the adaptation to osmotic shock plays a role for the type of incubations performed here [9, 10, 75]. The variable gene content among the congeneric species was especially enriched in glycosyltransferases and mobile elements, whose activity could have provided an ecological advantage during fluctuating environmental conditions, and thus resulted in replacing the previously dominant species [9, 10, 13], in addition to the action of any adaptive mutations in core (shared) genes*.* This genomic plasticity would also explain similar observations made in other systems such as the distinct temperature or light preferences of coexisting closely related taxa of *Curtobacterium* [66] or *Prochlorococcus* [7].

The persistent selection of prokaryotes with greater fitness also seemed to lead to the proliferation of their infecting viruses (VHRs >1) [76], thereby probably influencing the host abundances and possibly even some of the species’ replacement events mentioned above. Notably, the dynamics at the level of intraspecific sequence diversity were strongly correlated with the turnover in viral cohort diversity, pointing to the plausible control of strains and genomovars of specific key species by certain viral cohorts [77–80]. The temporal succession observed for distinct viral communities putatively infecting the same host indicated an important heterogeneity of viral genotypes over the course of the experiment [21, 58, 81, 82]. The consistent shifts in intraspecific diversity of the major species of both *Haloquadratum* and *Salinibacter*, as well as the diversity of their corresponding viral cohorts, indicated that the ecological succession of prokaryotic (host) species may be the result of the combined effects of fitness against the disturbances and control by their specific viral cohorts. Deciphering the relative importance of these two factors will require additional detailed, targeted experiments. The emergence of some latent viral genotypes that became abundant, and thus presumably active, with the increase in abundance of their respective hosts [77–80] could be considered as a plausible “ecosystem memory”, of viral cohorts surviving below detection levels of our sequencing efforts [83].

None of the observed shifts can be attributed to the microbiome of the tap water due to low cell numbers and biomass in that water, but also the fact that no known freshwater-derived organisms seem to survive and become abundant based on taxonomic identification of the recovered MAGs. We did observe a notable seasonal effect, especially over the two autumn–winter periods when the decrease in temperature and light intensity significantly lowered the evaporation rates. The prolonged periods between dilution events allowed part of the high-osmotic shock sensitive cells to increase their abundances before returning to lower abundance levels after the first spring dilutions.

It is true that for more robust conclusions, replicated mesocosms are desirable, which was, however, practically and/or economically unfeasible in our case given the large size of the mesocosms (230 L), the high frequency of sampling (weekly samples over 2 years), and the associated costs of sequencing and other molecular methods used (130 metagenomes were sequenced as part of the study). Further, and as reported elsewhere, replication may not always be necessary when the expected microbial community variability is small, and more frequent sampling of the series in such cases has more statistical power (and provides more biological insights) than a biological replicate of the series [25, 26, 84]. In our case, MASH distance variability between consecutive samples in each experiment (0.032 ± 0.002) was smaller than the largest difference (0.065) observed for samples from a control pond (no treatment) during a period of one month of natural development [6, 10], and the largest differences (0.057 for D20 and 0.07 for D13) were similar to that of the control pond, further justifying our choice to not establish replicate mesocosms.

Altogether, our results show that brine communities evolve in parallel to disturbances, and the resulting transitions seem to depend on the intensity of the perturbations. In all cases, we observed the communities transitioning to alternative stable states caused by reorganization in the community composition [1, 2, 85–89], mostly driven by species replacement within the same genus that were presumably functionally redundant, but with better fitness under the pressures applied. The low variability observed between samples and throughout the experiment suggests that hypersaline ecosystems are ecologically resilient (contrary to engineering resilience, [1, 2]) as the pre- and post-disturbance community structures seem not to functionally differ substantially.

The relatively simple community structure of brines near salt saturation proved to be an excellent model for ecological studies using controlled mesocosm experiments, similar to previous studies [13]. Our results show that the category of genus should be taken as a key unit in ecological studies and as an ecologically important level of diversity organization, and not just as a taxonomic rank. Further, the persistence and success of the genus level of diversity seem to rely on the genetic repertoire of the coexisting congeneric species due to relatively high functional overlap (gene sharing), which has broader implications for many molecular ecology studies. Also, we show that environmental pressures promote variations in species abundances that are mirrored by intraspecific shifts of their specific viral cohorts that probably control them along the time-series.

### Sampling permits

Brines were collected from S’Avall solar saltern, located in the south of the island of Mallorca (39°19′28″N; 2°59′21′′E), Spain, on 10 June 2020, and were authorized by the Spanish Ministry of Ecological Transition, with the permit numbers: ESNC22 and ESNC27.

## Supplementary Material

Suppl_Material_wrae215

Suppl_Tables_wrae215

## Data Availability

The datasets generated during the current study are available in the European Nucleotide Archive (ENA) repository at https://www.ebi.ac.uk/ena/browser/home, under BioProject accession number PRJEB75750.
