# Supplementary material for: Ecological success of extreme halophiles subjected to recurrent osmotic disturbances is primarily driven by congeneric species replacement
Source: ISME J. 2024 Oct 23;18(1):wrae215. doi: 10.1093/ismejo/wrae215 (PMC11544370; doi:10.1093/ismejo/wrae215)
Supplement: Suppl_Material_wrae215 [file suppl_material_wrae215.docx]

**Ecological success of extreme halophiles subjected to recurrent osmotic disturbances is primarily driven by congeneric species replacement**

Esteban Bustos-Caparros^1^*, Tomeu Viver^1,2^, Juan F. Gago^1^, Luis Miguel Rodriguez-Rojas^3,4^, Janet K. Hatt^5^, Stephanus N. Venter^6^, Bernhard M. Fuchs^2^, Rudolf Amann^2^, Rafael Bosch^1, 7^, Konstantinos T. Konstantinidis^5^, Ramon Rossello-Mora^1^*.

^1^ Marine Microbiology Group (MMG), Department of Animal and Microbial Biodiversity, Mediterranean Institute for Advanced Studies (IMEDEA, CSIC-UIB), Esporles, Spain.

^2^ Department of Molecular Ecology, Max Planck Institute for Marine Microbiology, Bremen, Germany.

^3^ Department of Microbiology, University of Innsbruck, Innsbruck, Austria.

^4^ Digital Science Center (DiSC), University of Innsbruck, Innsbruck, Austria.

^5^ School of Civil and Environmental Engineering, Georgia Institute of Technology, Atlanta, GA, USA.

^6^ Department of Biochemistry, Genetics and Microbiology, and Forestry and Agricultural Biotechnology Institute (FABI), University of Pretoria, Pretoria, South Africa.

^7^ Microbiologia, Departament de Biologia, Edifici Guillem Colom, Universitat de les Illes Balears, Campus UIB, 07122 Palma de Mallorca, Spain.

*Corresponding authors: Esteban Bustos-Caparros, Marine Microbiology Group (MMG), Department of Animal and Microbial Biodiversity, Mediterranean Institute for Advanced Studies (IMEDEA, CSIC-UIB), Carrer Miquel Marquès 21, Esporles, Illes Balears, 07190, Spain. Email: [ebustos@imedea.uib-csic.es](mailto:ebustos@imedea.uib-csic.es), Ramon Rossello-Mora, Marine Microbiology Group (MMG), Department of Animal and Microbial Biodiversity, Mediterranean Institute for Advanced Studies (IMEDEA, CSIC-UIB), Carrer Miquel Marquès 21, Esporles, Illes Balears, 07190, Spain. Email: [ramon@imedea.uib-csic.es](mailto:ramon@imedea.uib-csic.es)

**Supplementary Figure S1:** Experimental mesocosms D13 and D20. A) saturated brines just before the first dilution event (22^th^ June 2020; 36% salts), B) after the first dilution event (23^th^ June 2020; 13% and 20% salts), and C) time-813 days (1^th^ September 2022; 36% salts). D) Cellular biomass collected from time-813 days. Brownish (left) and reddish (right) cells corresponded to D13 and D20 mesocosms, respectively. E) Sampling schema of D13 and D20 mesocosms and the number of osmotic cycles.


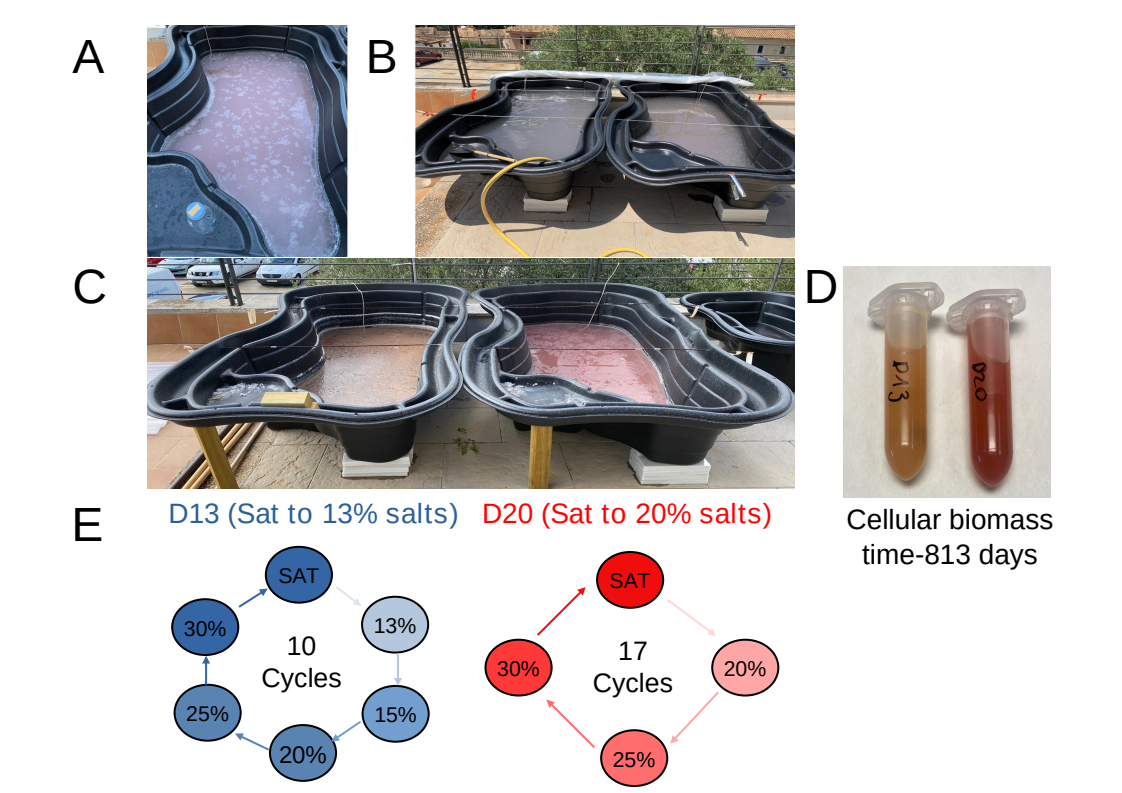


**Supplementary Figure S2:** A) Google Earth satellite image of D13 and D20 mesocosms located outside the Institute exposed to natural environmental conditions with little shading from surrounding mountains, buildings or trees. B) D13 and D20 mesocosms covered with transparent polycarbonate corrugated sheets used for roofing to prevent dilution of brine concentration by rain.


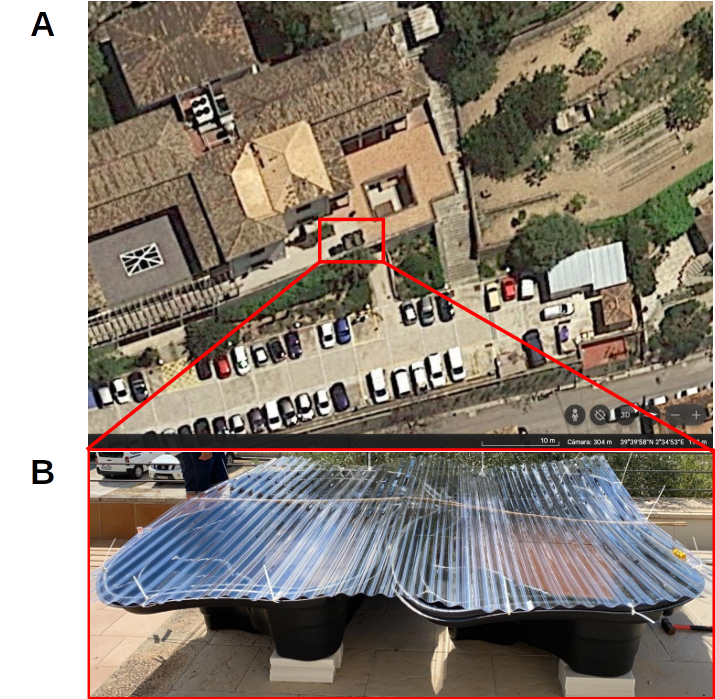


**Supplementary Text T1: Experimental setup**

A total of 640L of brines were collected from S’Avall solar saltern located in the south of the island of Mallorca (39°19′28″N – 2°59′21′′E), Spain (10^th^ June 2020). Brines were transported to the IMEDEA facilities (Esporles, Spain) using 50L plastic cans previously sterilized with 70% ethanol. At IMEDEA, brines were homogenized and subsequently divided in two equal volumes of 320L and stored in two contiguous rigid plastic ponds (typically employed in gardening), each with a capacity of 1000L and with an irregular shape (Supp. Figure S1). The ponds were located one next to the other, left outside the institute, exposed to the sunlight with little shading from surrounding mountains, buildings or trees, and left exposed to the natural environmental conditions except for rainfall (Supp. Figure S2A). To prevent dilution from rainfall, both mesocosms were covered with transparent polycarbonate corrugated sheets used for roofing, but only when the weather forecast indicated rain (Supp. Figure S2B). Brines with an initial salinity of 25.5% were left evaporating, and when NaCl saturation was reached (>36% NaCl at 25ºC; [1]), each pond was subjected to one of two distinct treatments: one was cyclically diluted from saturation to 13% salinity (D13 mesocosm) and the other to 20% salts (D20 mesocosm). To dilute the brines, we used tap water (TW) with a flow rate of 25 L/minute and continuous mixing with a plastic, disinfected oar. Salinity and temperatures were regularly monitored using a Sper Scientific Salt Refractometer and a HOBO Water Temp Pro v2 devices, respectively. Samples were collected 24 hours after each dilution event and also when salinity reached 15%, 20%, 25%, 30% salts, and upon salt saturation. The nomenclature of the samples is *Mesocosm* (D13 or D20)*_Sampling Julian day_Salinity* (e.g. D13_12_36 is pond D13, at day 12^th^ with a salinity of 36%; Supp. Table S1). This experiment was conducted over two years and almost three months, precisely, 813 days. Finally, the ponds were left at saturation for a long-term response over an additional period of one year with no other pressures than those occurring naturally. The metagenomes of the additional 5 samples covering the period of days 1055 to 1178 were only used to evaluate the metagenome differences using MASH distance in Figure S6, but not further investigated in this disturbance study.

**REFERENCES**

[1]. Haynes WM, Lide DR, Bruno TJ. *CRC handbook of chemistry and physics*. Boca Raton, USA, 2016. <https://doi.org/10.1201/9781315380476>.

**Supplementary Text T2: Phylogenetic 16S rRNA analysis based on OPUs**

Reads associated with 16S rRNA genes were extracted using Parallel-META v.2.4 [1] and clustered into Operational Taxonomic Units (OTUs) at ≥ 98.7% identity using the *pick_closed_reference_otus.py* script from QIIME tool [2]. The representative sequence (longest) of each OTU was aligned with SINA tool [3] and added by parsimony, both implemented in the ARB tool [4], to the pre-built reference database SILVA REF138 [5]. OTUs were checked and grouped manually into Operational Phylogenetic Units (OPUs) as previously detailed [6].

**REFERENCES**

[1]. Su X, et al. Parallel-META 2.0: enhanced metagenomic data analysis with functional annotation, high performance computing and advanced visualization. *PloS One* 2014; **9**: e89323.

[2]. Caporaso JG, et al. QIIME allows analysis of high-throughput community sequencing data. *Nat Methods* 2010; **7**: 335-336.

[3]. Pruesse E, et al. SILVA: a comprehensive online resource for quality checked and aligned ribosomal RNA sequence data compatible with ARB. *Nucleic Acids Res* 2007; **35**: 7188-7196.

[4]. Ludwig W, et al. ARB: a software environment for sequence data. *Nucleic Acids Res* 2004; **32**: 1363-1371.

[5]. Quast C, et al. The SILVA ribosomal RNA gene database project: improved data processing and web-based tools. *Nucleic Acids Res* 2013; **41**: D590–D596.

[6]. Mora-Ruiz MDR, et al. Moderate halophilic bacteria colonizing the phylloplane of halophytes of the subfamily *Salicornioideae* (*Amaranthaceae*). *Syst Appl Microbiol* 2015; **38**: 406-416.

**Supplementary Text T3 mesocosm preparation and initial features:**

Brines from S’Avall were collected at a salinity of 25.5% (Figure 1C; Supp. Table S1) and contained Cl^-^, Na^+^, Mg^2+^ and SO_4_^2-^ as the main ions measured (Supp. Table S1; Supp. Figure S3). Brines contained 1.6x10^8^ ± 5.3.10^6^ cells/ml with a strong dominance of Archaea (1.1x10^8^ ± 3.2x10^6^ cells/ml; 71.42% of the total cell counts) over Bacteria (4.5x10^7^ ± 3.2x10^6^ cells/ml; 28.58% of the total cell counts) (Figure 1D and E; Supp. Table S1). After 12 days evaporation (22^th^ June 2020), both mesocosms reached salt-saturation (>36% salts), and the cell counts increased to ~2.52x10^8^ ± 8.5x10^6^ cells/ml (Figure 1D; Supp. Table S1), and the relative archaeal and bacterial proportions remained similar (~66% and ~34%, respectively; Figure 1E; Supp. Table S1).

**Supplementary Text T4: testing the influence of tap water microbiology in the experiment**

The ionic concentration of the inflow tap water (TW) was 1200 times lower than the saturation brines, mainly dominated by sodium chloride and sulfate. Proportions of these ions were maintained in the mesocosms after dilution (Supp. Table S1). Altogether we could not see any significant variation in the ionic composition (Supp. Figure S3; Supp. Table S1) throughout the experiment. To ensure that the addition of tap water (TW) did not strongly influence the brine microbial composition beyond a dilution effect, we first calculated that its microbial cell abundance (DAPI) was ~2.71x10^5^ ± 1.86x10^4^ cells/ml (Supp. Table S1). Then, we added ~3.8x10^7^ and ~8.6x10^7^ cells in each dilution event in D13 and D20 respectively. These values were six orders of magnitude below the total cells present in the total volume of original brines calculated to be 2.4x10^13^ ± 4.8x10^10^ cells in each pond. In addition, MASH distances among the 131 sequenced metagenomes (TW, time-zero, 59 from D13, and 70 from D20) using Non-metric Multidimensional Scaling (NMDS) showed strong metagenome dissimilarity of TW compared to D13 and D20 metagenomes (Supp. Figure S4). The recruitment of the assembled TW contigs of D13 and D20 reads was lower than 0.001%, again indicating a lack of potential influence of the tap water on the microbial composition of the mesocosm. We cannot disregard a potential, but undetected, influence of the added water on the chemical and microbial composition, but given the very low cell numbers and proportion of ions added, we believe that any effect is likely to be minimal.

**Supplementary Figure S3:** Temperature (ºC), salinity (%), and ionic composition (g/L) over 813 days in D13 and D20 mesocosms.


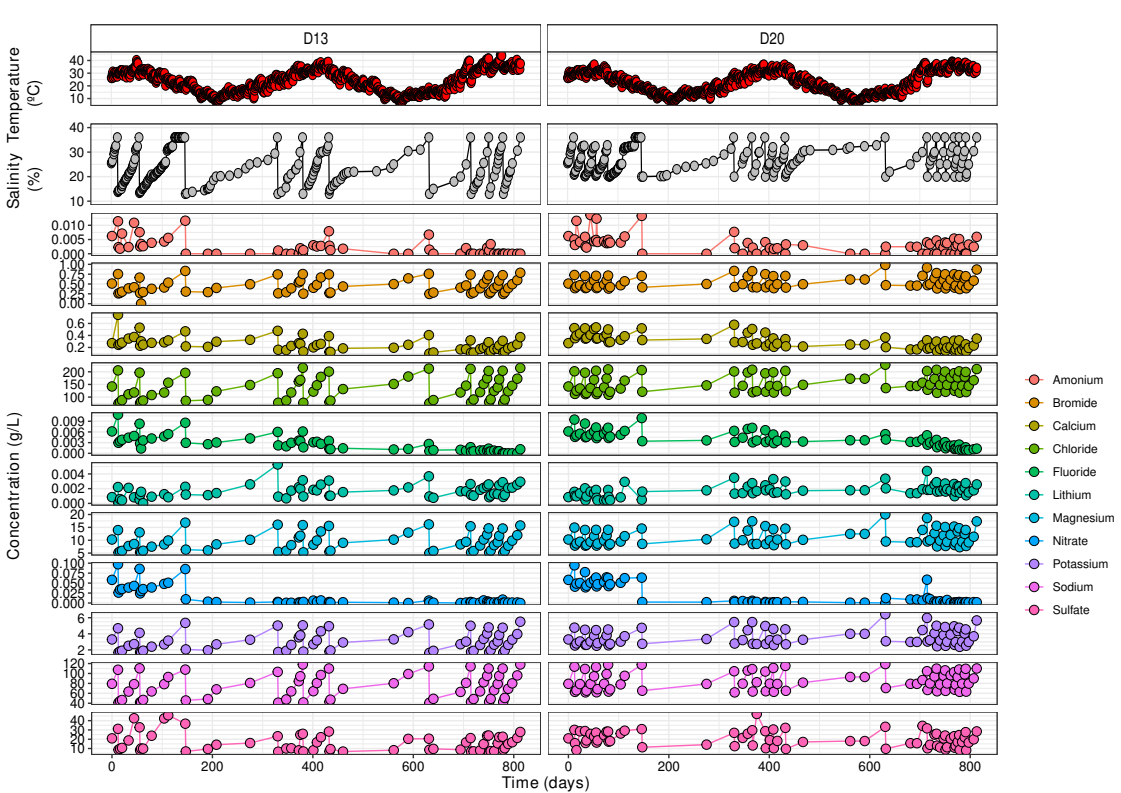


**Supplementary Figure S4:** NMDS plot based on MASH distances using all D13, D20, and TW metagenomes.


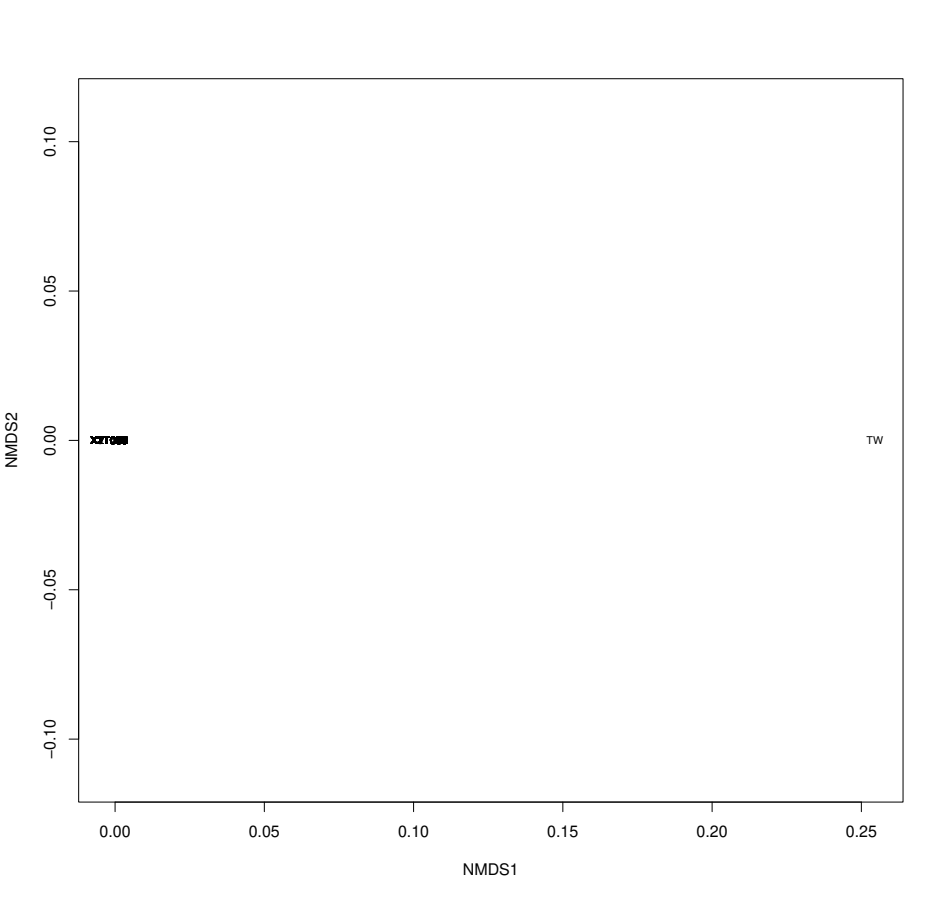


**Supplementary Figure S5: (A)** Temperature (ºC), (**B)** salinity (%), and (**C)** percentage of resistant bacterial (green) and archaeal (red) cells after every dilution event in D13 and D20 mesocosms across 813 days.

​
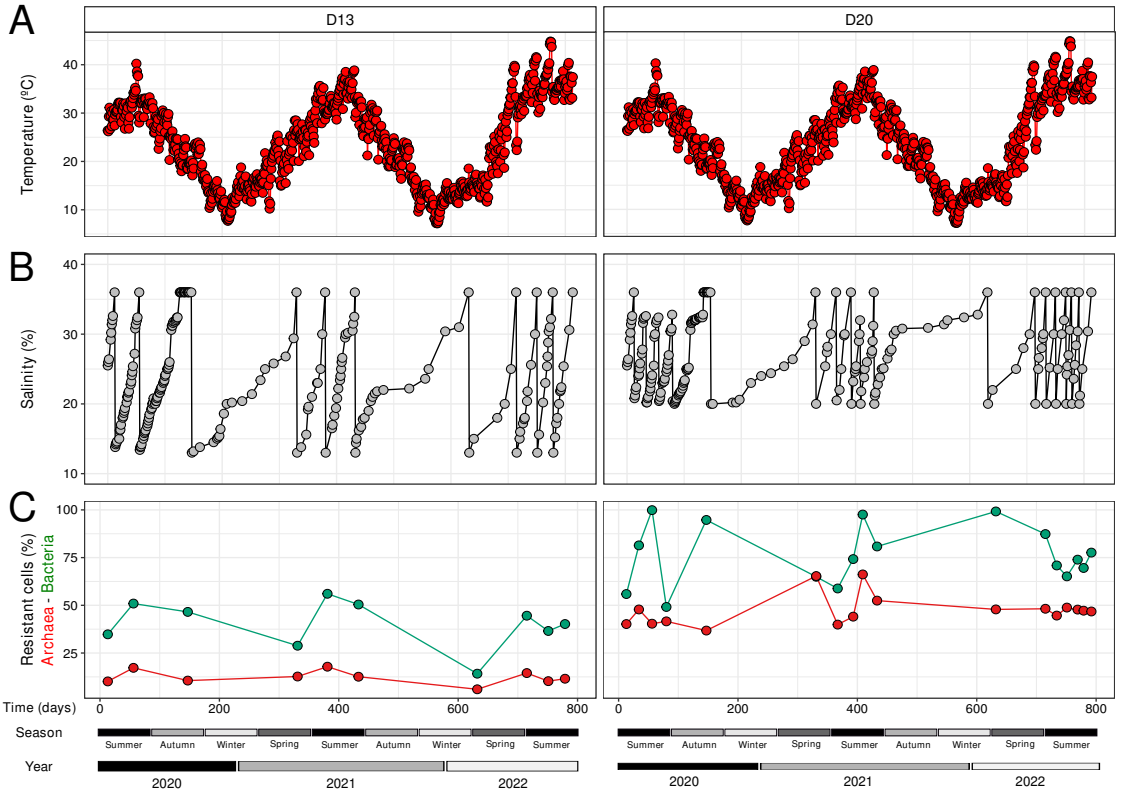
​​​

**Supplementary Text T5: Microbial and viral diversity of D13 and D20 mesocosms over the 813 days of the disturbance experiment.**

From the 130 sequenced metagenomes (excluding the TW metagenome from the analysis), we recovered a total of 2,745 MAGs from time-zero (34 MAGs), D13 (1,293 MAGs) and D20 (1,418 MAGs), rendering 128 species groupings of MAGs at an ANI threshold ≥95% (Table S4). Additionally, we identified 535 OPUs, for which 208 and 327 affiliated with Bacteria and Archaea, respectively (Table S5). Based on MIMAG standards [1], 35.94% of the species were classified as high-quality and 64.06% as medium-quality MAGs. Taxonomic affiliations indicated that members of the Archaea were classified as 3, 10 and 70 distinct species of *Nanoarchaeota*, *Nanohaloarchaeota,* and *Halobacteriota*, respectively (Table S4). Among the *Halobacteriota* species the most representative order was *Halobacteriales* that comprised four distinct families: *Halococcaceae* (n=1), *Saliniarchaeaceae* (n=1), *Haloarculacea* (n=29), and *Haloferacaceae* (n=37), with *Halobellus*, *Natronomonas,* and *Halorubrum* genera with the highest number of distinct species (Table S4). On the other hand, for Bacteria we detected 7 distinct phyla, with *Cyanobacteria* (n=4), *Proteobacteria* (n=11), and *Bacteroidota* (n=22) having the most representatives (Table S4). We detected a great number of species within the *Salinibacteraceae* family (n=17) with 9 species assigned to the *Salinibacter* genus (Table S4). Only 10 species (7.75%) were identified as members of known cultured species (Table S4), and 60% of the bacterial and 71.1% of the archaeal species were identified as members of known cultivated genera, but yet unclassified species (Table S4).

We identified 38,056 putative viral contigs that ranged between 5 – 206 kb (Table S6) for which 79.28% could be taxonomically classified to *Caudoviricetes* (Table S6). Based on an ANI thresholds ≥95% and alignments ≥85%, we identified 7,451 distinct vOTUs for which 67.25% was assigned to our MAGs and/or available genomes (Table S7), and 47.82% of them could be assigned to 97 species extracted from our metagenomic dataset (Table S7). Consistent with the host taxonomy, the majority of vOTUs were assigned to *Proteobacteria* (n=249), *Bacteroidota* (n=1,036), and *Halobacteriota* (n=3.214), with *Halorubrum* (n=371), *Halonotius* (n=416), *Salinibacter* (n=584), and *Haloquadratum* (n=1,260) the microbial hosts with the most assigned vOTUs (Table S7).

**REFERENCES**

[1]. Bowers RM, et al. Minimum information about a single amplified genome (MISAG) and a metagenome-assembled genome (MIMAG) of bacteria and archaea. *Nature biotechnology*, 2017; **35**, 725-731.

**Supplementary Figure S6:** Bubble plot showing the relative abundances (%) of microbial species over time in the D13 and D20 mesocosms.


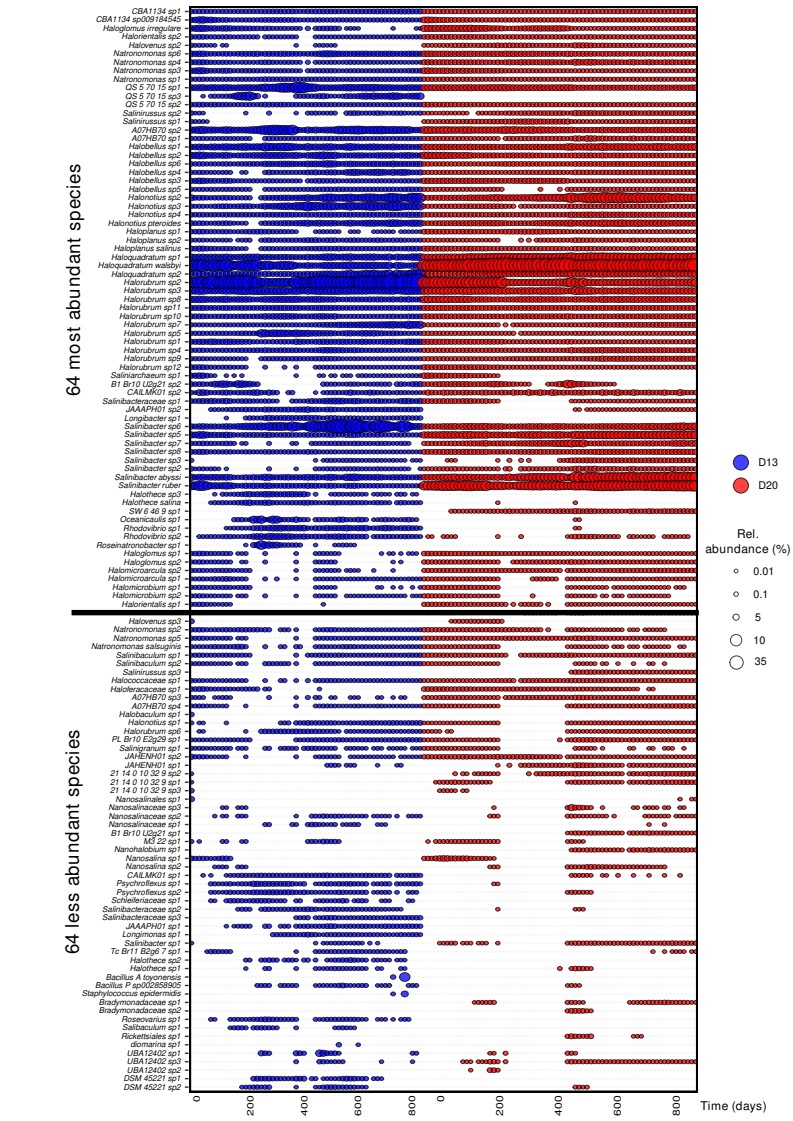


**Supplementary Figure S7:** Barplot of the Linear Discriminant Analysis (LDA) scores showing the statistically differential preferences of microbial (MAGs) and viral (vOTUs) species at genus level between both mesocosms using non-parametric Kruskal-Wallis (KW) sum-rank test. Black-dashed lines indicated scores > 2.


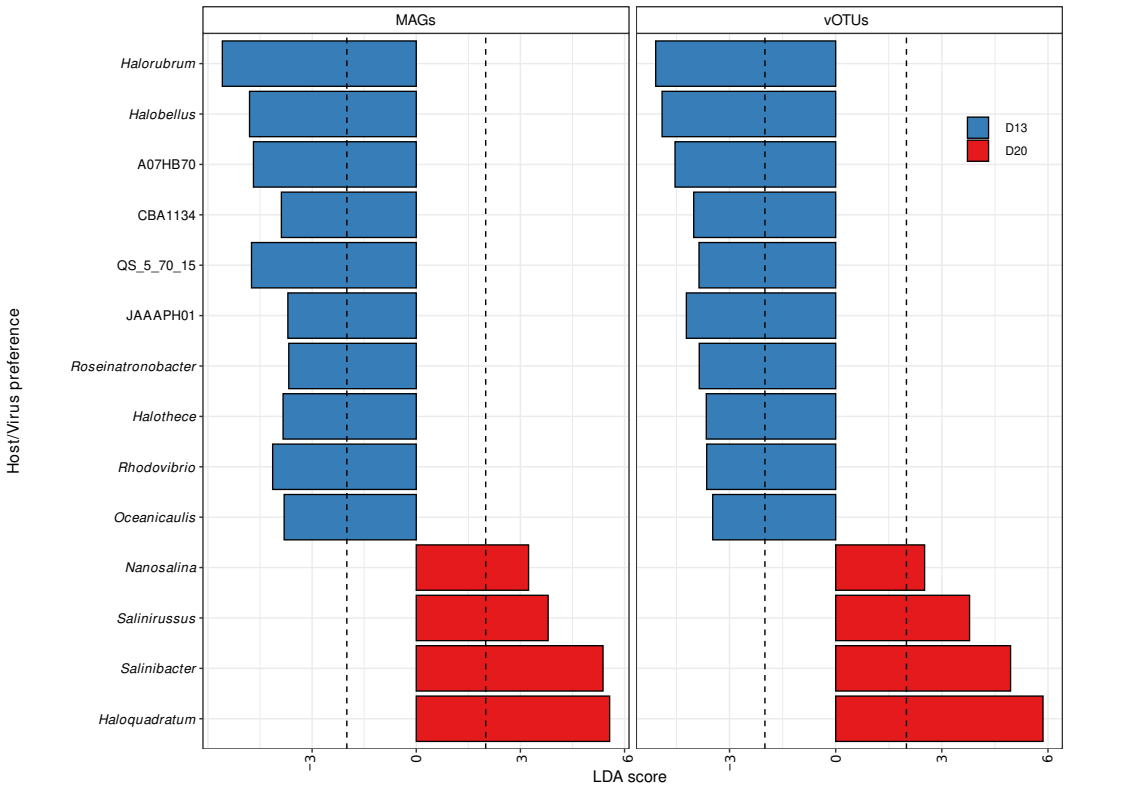


**Supplementary Figure S8:** Boxplots representing the species diversity (Shannon H’) comparison between D13 and D20 mesocosms for archaeal, bacterial, and viral fractions.

**
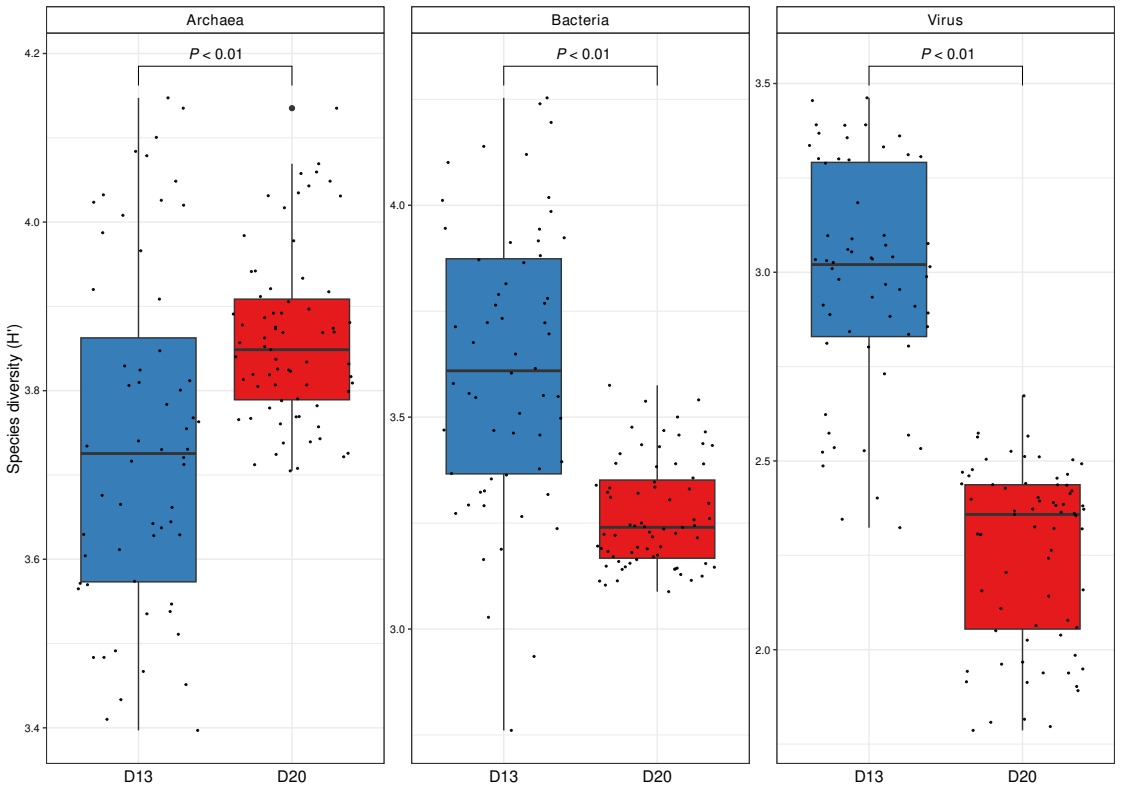
**

**Supplementary Figure S9: (A)** Temperature (ºC), and **(B)** Functional diversity in D13 and D20 mesocosms over 813 days. **(C)** Boxplot based on functional diversity (H’) between D13 and D20 mesocosms using KOs abundances.

**
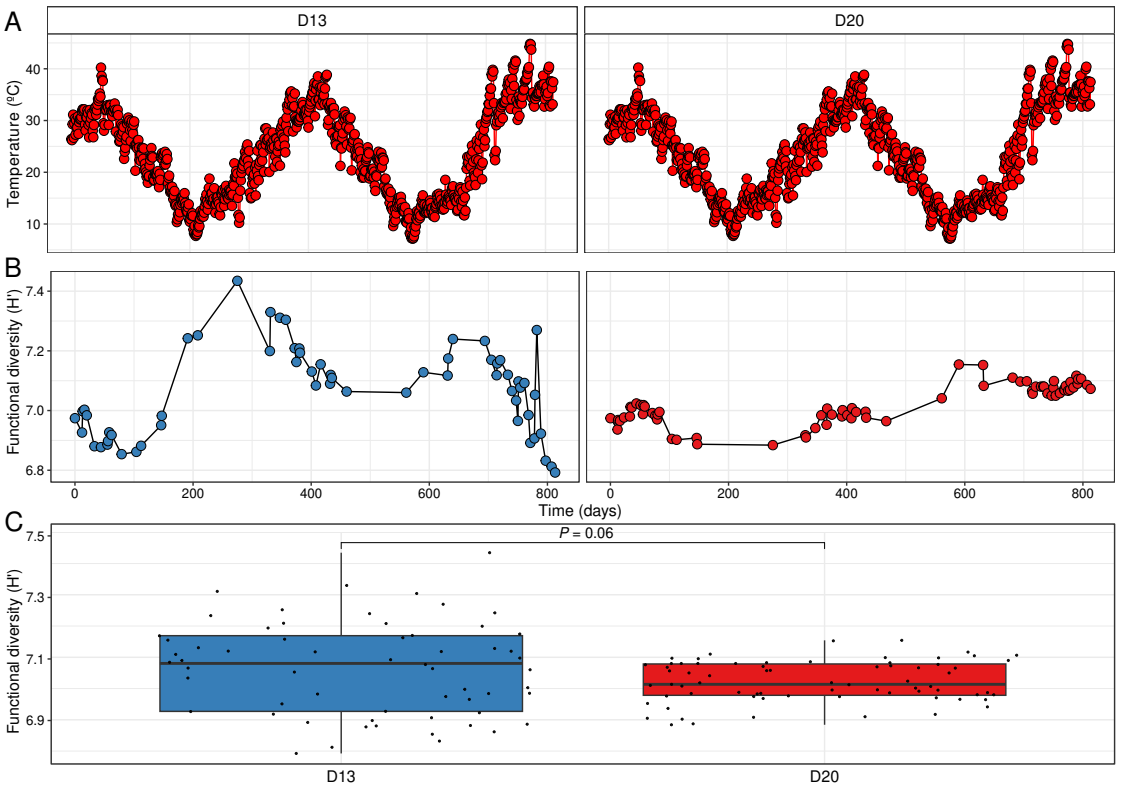
**

**Supplementary Figure S10:**  Relative abundance of the 14 genera (excluding *Haloquadratum, Halorubrum,* and *Salinibacter* members illustrated in Figure 3) with at least two coexisting species in the D13 and D20 metagenomes (n=130). The relative abundance of each genus was estimated calculating the sequencing effort (sequencing depth divided by number of metagenomic reads).


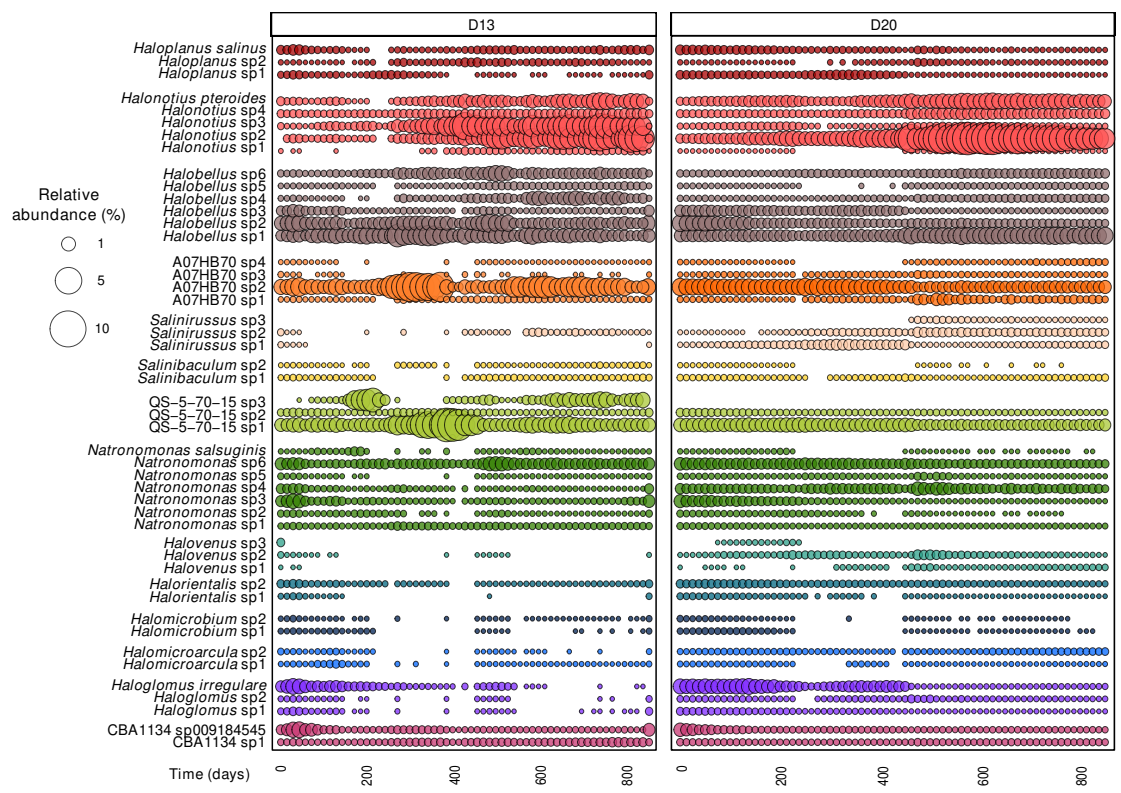


**Supplementary Figure S11:** Phylogenetic reconstruction using the concatenated core orthologous proteins extracted from the type-strain genomes and the representative (highest quality) MAGs of **A)** *Halorubrum, Haloquadratum,* and **B)** *Salinibacter* species. Both phylogenetic trees were reconstructed with a Neighbor Joining algorithm using the kimura correction. The bar indicates a 10% sequence divergence for each tree. **C)** Functional redundancy of type-strain core proteins of *Haloquadratum, Halorubrum,* and *Salinibacter* species. Heatmap representing the main metabolic pathways inferred for the core (i.e., shared >90% species) and auxiliary (i.e., shared <90% species) proteins based on the presence or absence.


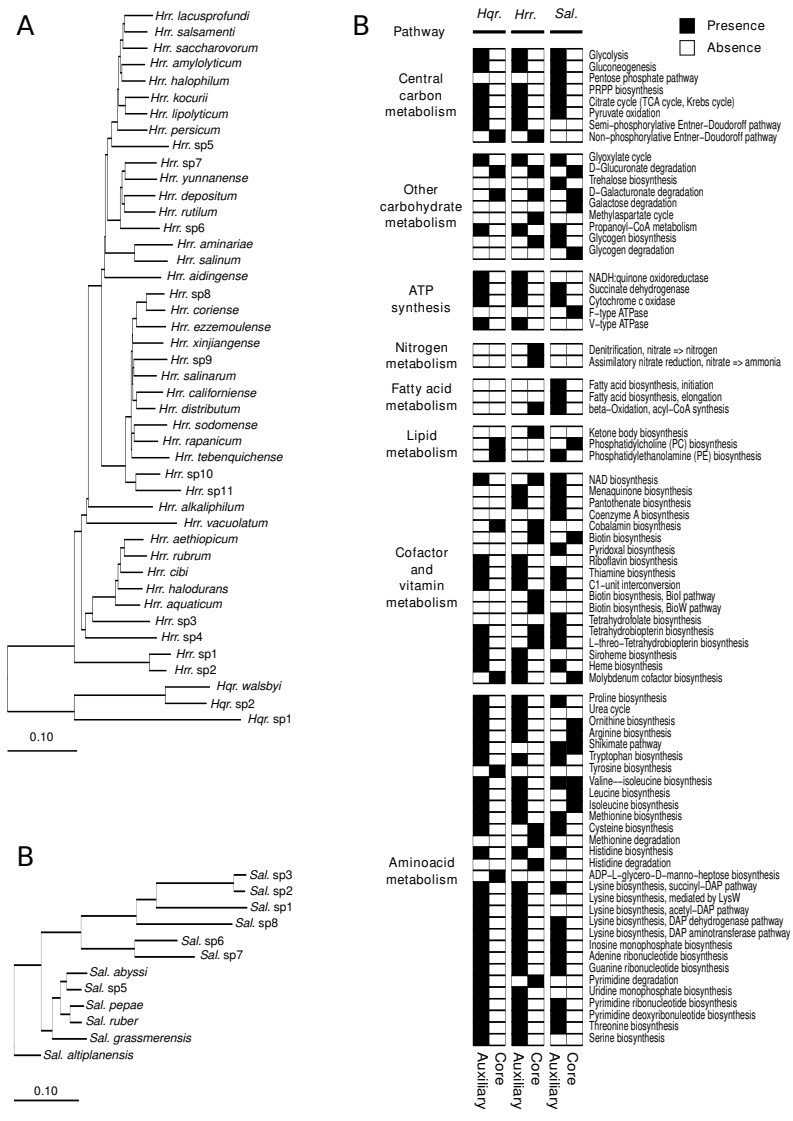


**Supplementary Figure S12:** Barplots representing the functional annotation of non-redundant proteins clustered at ≥50% identity and ≥50% identity comparing **A)** *Haloquadratum* sp2 vs. *Hqr. walsbyi* and **B)** *Salinibacter* sp6 vs. *Sal. ruber* using COG categories extracted from the COGclassifier tool.

**
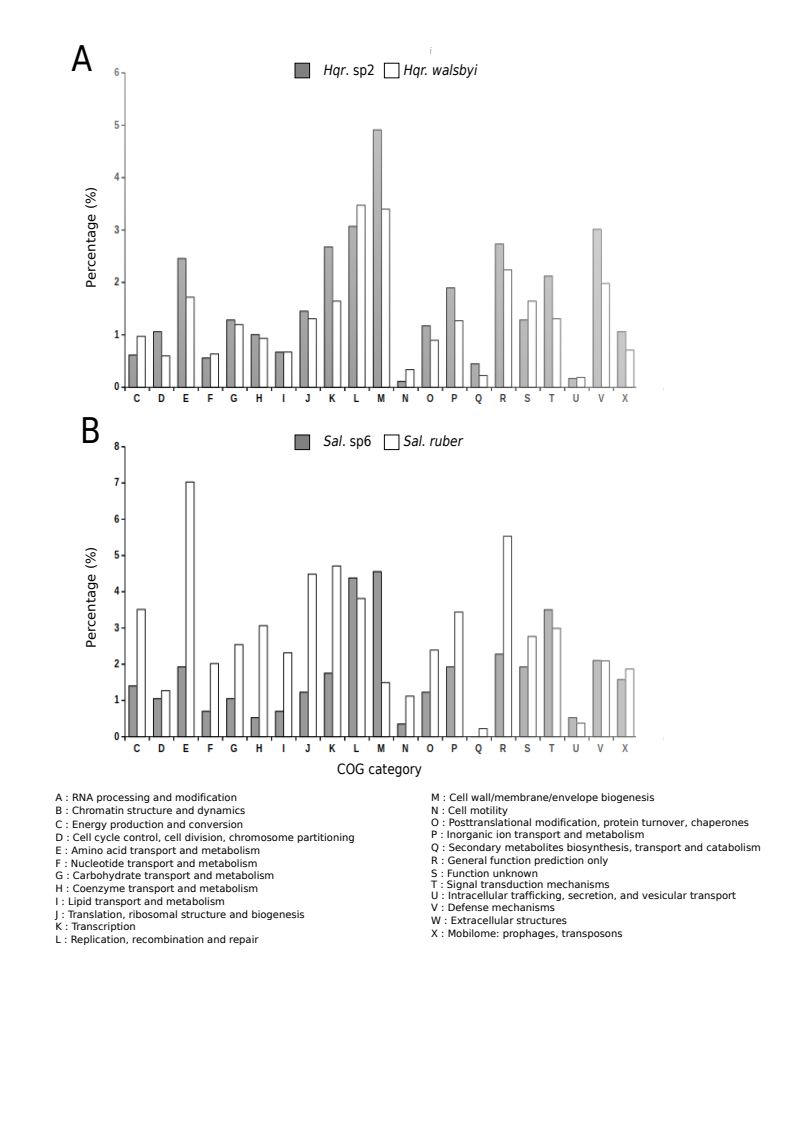
**

**Supplementary Figure S13:** Lineage-specific viral-host abundance ratios (VHRs) of D13 (top) and D20 (bottom) mesocosms. Dashed lines indicate VHR = 1 and asterisks show in which mesocosms species have statistically higher values of VHRs.


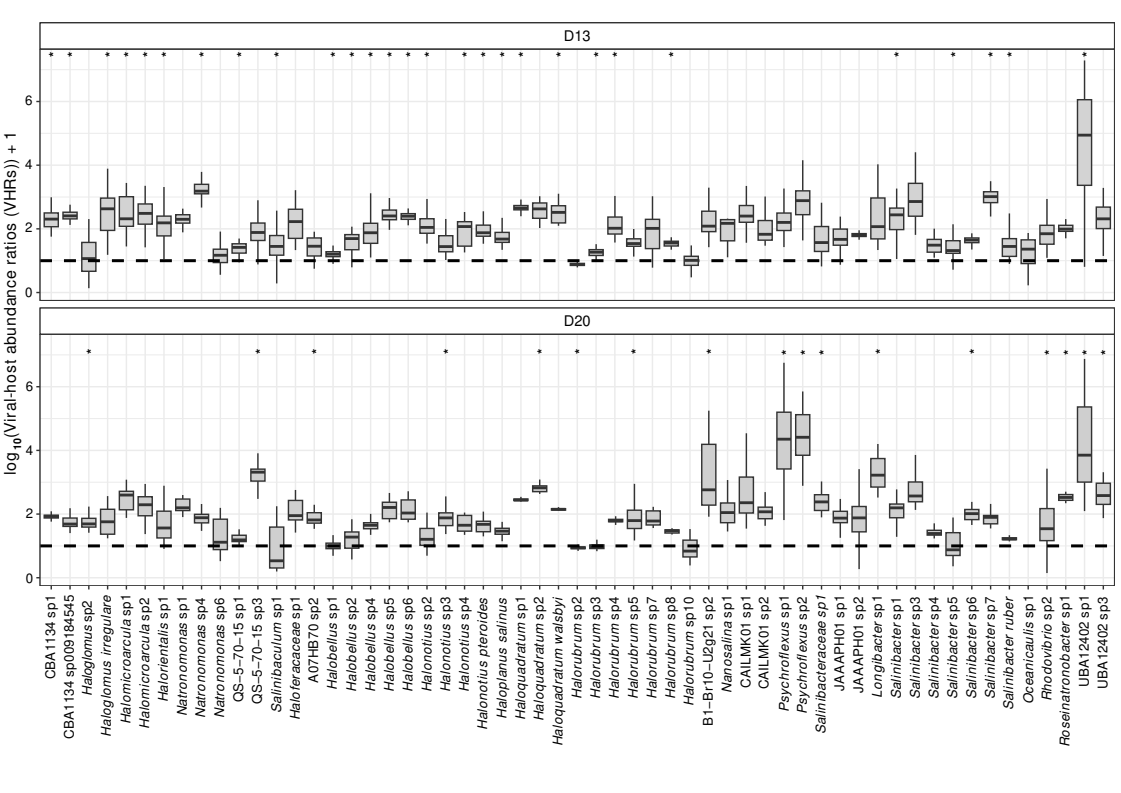


**Supplementary Figure S14:** Boxplots based on viral-host abundance ratios (VHRs) between D13 and D20 using **A)** all, **B)** Archaea, and **C)** Bacteria species.

**
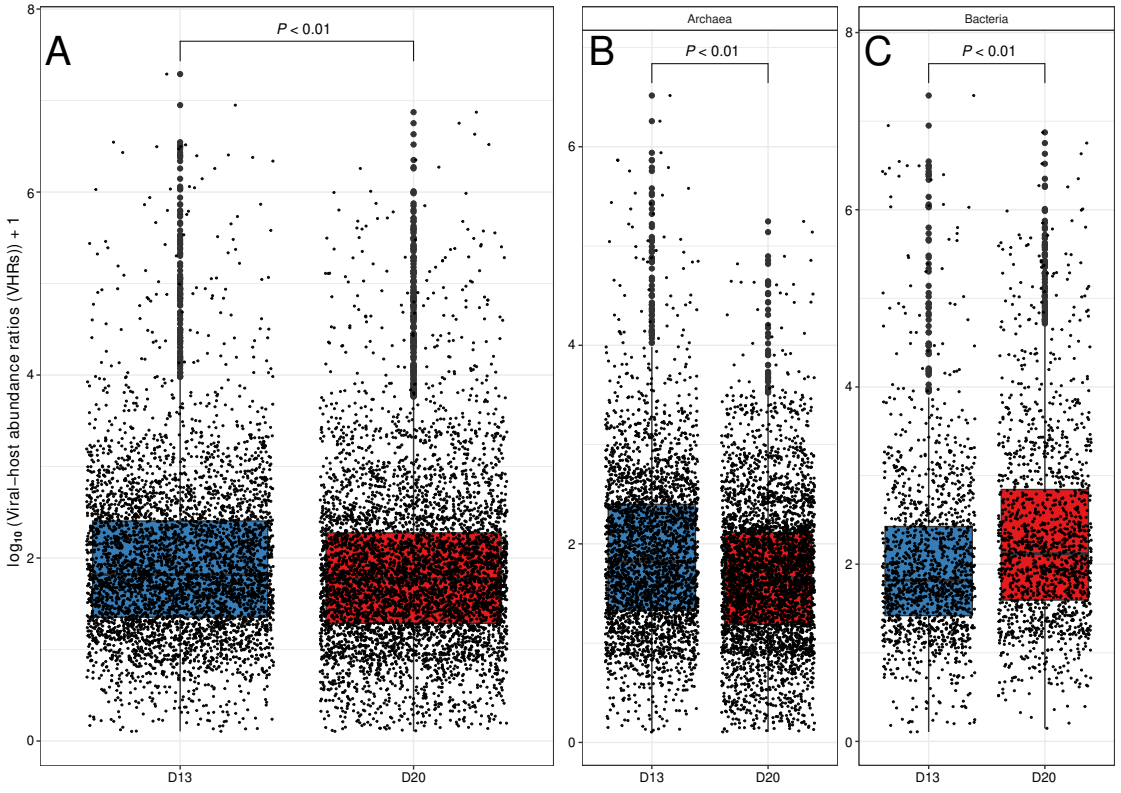
**

**Supplementary Figure S15:** Examples of heatmaps representing the temporal succession of viral communities based on abundance of vOTUs infecting **A)** *Hqr. walsbyi****,* B)** *Haloquadratum* sp2**, C)** *Sal. ruber,* and **D)** *Salinibacter* sp6. vOTUs abundances were calculated based on sequencing effort (sequencing depth divided by number of metagenomic reads) across metagenomes (n=130).

**
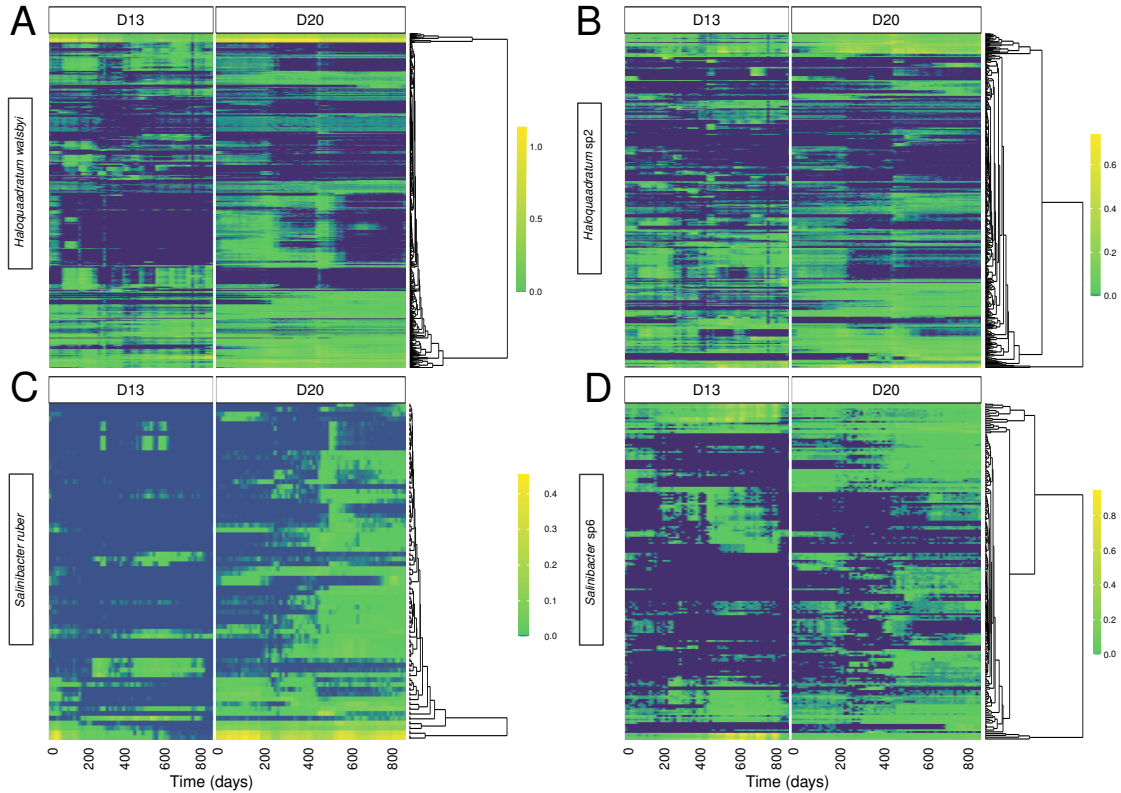
**
